# Admission antithrombin III activity as a risk-enrichment marker for septic shock below the ISTH overt-DIC threshold

**DOI:** 10.3389/fmed.2026.1899792

**Published:** 2026-07-31

**Authors:** Tao Zhou, Wanying Cheng, Guohua Jiang, Xiaojin Zhang, Li Liu, Jun Wu

**Affiliations:** 1 The First Clinical Medical College of Nanjing Medical University, Nanjing, China; 2 Department of Geriatrics, Jiangsu Provincial Key Laboratory of Geriatrics, The First Affiliated Hospital of Nanjing Medical University, Nanjing, China; 3 Department of Hematology, Wuxi People's Hospital Affiliated to Nanjing Medical University, Wuxi Medical Center, Wuxi, China

**Keywords:** antithrombin III, biomarker, diagnostic discrimination, disseminated intravascular coagulation, ISTH DIC score, septic shock

## Abstract

**Background:**

International Society on Thrombosis and Haemostasis (ISTH) overt disseminated intravascular coagulation (DIC) scoring is a standard bedside framework for infection-associated coagulopathy but does not capture anticoagulant-pathway markers. The study examined whether admission antithrombin III (AT-III) activity provided risk-enrichment information for septic shock among adults admitted with infection below the ISTH 2001 overt-DIC threshold.

**Methods:**

The study retrospectively analyzed 1,286 adults admitted with infection. AT-III activity was evaluated in the ISTH-complete and below-threshold subsets and compared with ISTH 2001 and 2025 SSC overt-DIC scores. Retrospective temporal adjudication compared first valid AT-III report availability with operational septic-shock onset. In a clinically indicated lactate-measured subset, the study assessed whether AT-III added discrimination beyond age, sex, and lactate. Firth-penalized regression evaluated the conditional association of AT-III with septic shock beyond age, sex, and raw ISTH components.

**Results:**

Among 1,027 ISTH-complete patients, 974 (94.8%) were below the ISTH 2001 overt-DIC threshold, including 44 of 60 septic-shock cases. In this subgroup, 43 of 44 shock cases had AT-III activity <80%; the area under the curve (AUC) was 0.844 and the positive predictive value was 9.37% (43/459). Of 82 septic-shock admissions, 74 were temporally adjudicable: 62 were incident and 12 were prevalent or concurrent at AT-III report availability. In incident cases, AT-III report availability preceded operational shock onset by a median of 7.6 h (interquartile range (IQR) 5.0–9.6); sampling therefore also preceded onset. In the lactate-measured subset (*n* = 518), AT-III increased AUC beyond age, sex, and lactate from 0.740 to 0.821; this increment persisted in the incident-shock analysis (0.680–0.792). In the primary Firth model, lower AT-III activity remained conditionally associated with shock (OR 3.10 per 1-SD decrease); the apparent and bootstrap-corrected AUCs were 0.900 and 0.888.

**Conclusion:**

Admission AT-III activity was associated with septic shock within the large ISTH below-threshold infection subgroup and provided high-sensitivity, low-positive-predictive-value enrichment information. Retrospectively reconstructed temporal ordering demonstrated a report-release-to-operational-onset interval in incident cases but does not establish prospective prediction, prospective workflow performance, or clinical benefit. AT-III should not be used as a standalone trigger for escalation decisions. Prospective, time-stamped validation is required.

## 1 Introduction

Sepsis, defined as life-threatening organ dysfunction caused by a dysregulated host response to infection (1), carries a substantial global burden (2, 3), and disseminated intravascular coagulation (DIC) is the most severe expression of sepsis-associated coagulopathy (4, 5). The International Society on Thrombosis and Haemostasis (ISTH) overt-DIC score (6) remains a widely used bedside and research framework for grading consumption coagulopathy. Although the Surviving Sepsis Campaign 2021 guidance (7) addresses sepsis management broadly, an admission-stage clinical problem remains: how to recognize coagulation-related deterioration before overt-DIC criteria are met. The 2025 ISTH SSC update revised the definition and scoring criteria for overt DIC (8).

Both the 2001 and 2025 ISTH frameworks rely primarily on platelet count, coagulation-time prolongation, fibrinogen, and fibrin-related markers. These variables capture platelet consumption, procoagulant activation, and fibrinolytic disturbance, but they do not directly measure the endogenous anticoagulant pathway (9–12). Consequently, an overt-DIC score may remain below threshold even when the anticoagulant limb of sepsis-associated coagulopathy is impaired. Because overt-DIC frameworks are designed to classify DIC rather than septic shock, a below-threshold score in a patient with septic shock should not be interpreted as a failure of ISTH-based DIC assessment. Antithrombin III (AT-III), the principal endogenous inhibitor of thrombin and factor Xa (10), represents a complementary anticoagulant-pathway marker that may provide additional information in this below-threshold clinical space.

Experimental studies have suggested that neutrophil elastase can inactivate or contribute to depletion of antithrombin under inflammatory conditions (13–15), and high-dose AT-III suppressed lipopolysaccharide- and platelet-induced neutrophil extracellular trap formation *in vitro* (16). Clinical studies have linked reduced AT-III activity to sepsis severity, DIC, and adverse outcomes (17–20).

However, most have evaluated AT-III as a severity or prognostic marker in patients with established sepsis, sepsis-induced coagulopathy, or DIC, rather than asking whether an admission AT-III result provides clinically interpretable risk stratification among infected patients who do not meet overt-DIC criteria. Studies pairing AT-III activity with antigen are limited; such measurements may offer exploratory biological context but do not by themselves establish a mechanism of AT-III dysfunction. Most prior work also predates the 2025 ISTH SSC framework. Importantly, the clinical interpretation of an admission AT-III result depends on whether it becomes available before incident shock onset and whether it adds information alongside commonly used clinical markers, including lactate when clinically measured.

This study therefore examined whether admission AT-III activity provided risk-enrichment information for septic shock among adults admitted with infection, with emphasis on the subgroup below the ISTH 2001 overt-DIC threshold. AT-III activity was compared with the ISTH 2001 and 2025 SSC frameworks as contextual assessments for a septic-shock endpoint; evaluated its conditional association with septic shock beyond age, sex, and raw ISTH components; retrospectively adjudicated the temporal relationship between AT-III report availability and operational shock onset; and examined its discrimination alongside clinically indicated lactate. Additionally, the study explored an activity-to-antigen pattern as biological plausibility and evaluated direction consistency in a selected MIMIC-IV AT-III-tested subgroup. AT-III was interpreted as a complementary risk-stratification marker rather than a replacement for ISTH-based DIC assessment.

## 2 Materials and methods

### 2.1 Study design and data sources

This was a single-center retrospective diagnostic-accuracy cohort study conducted at the Affiliated Wuxi People’s Hospital of Nanjing Medical University. The discovery cohort comprised consecutive adults admitted with a primary diagnosis of infection between January 2023 and December 2025. Demographic, clinical-classification, treatment, and laboratory data were extracted from the hospital electronic information system and laboratory information system.

Clinical severity classification for the index hospitalization was determined independently by two trained abstractors through review of diagnostic and treatment records. Abstractors were blinded to AT-III activity and ISTH score calculations, and disagreements were adjudicated by a third reviewer (J. W.). The MIMIC-IV v3.1 database was used for a selected AT-III-tested cross-cohort direction-consistency analysis (Section 2.8).

Reporting followed the Strengthening the Reporting of Observational Studies in Epidemiology (STROBE) statement (21) and the Standards for Reporting Diagnostic Accuracy Studies 2015 (STARD 2015) guideline (22) (Supplementary Files 1–2). The study was approved by the Institutional Review Board of the Affiliated Wuxi People’s Hospital of Nanjing Medical University (approval number KYLL51308; 12 January 2023) and was conducted in accordance with the Declaration of Helsinki. The requirement for written informed consent was waived because the analysis used retrospective de-identified data.

### 2.2 Participants, eligibility criteria, and time zero

Inclusion criteria were: (1) age 18–70 years at admission according to the source-cohort eligibility framework; (2) a primary admission diagnosis of infection in the admission diagnostic record; and (3) availability of at least one admission coagulation test. Exclusion criteria were a primary diagnosis not classifiable into one of four prespecified clinical severity groups, known inherited coagulation disorders, or therapeutic anticoagulation at admission.

For the index hospitalization, patients were classified into four mutually exclusive clinical severity groups according to the highest severity reached during that admission: infection/fever, bacteremia without Sepsis-3 criteria, Sepsis-3 sepsis, and septic shock. Thus, patients who developed septic shock after admission were classified in the septic-shock group for severity-stratified analyses.

Time zero was hospital admission. The baseline measurement window, w0, was defined as the first 24 h after admission and was used to identify baseline AT-III activity and admission laboratory measurements required for ISTH scoring and clinical models. The w0 definition did not require septic shock to be present at admission.

### 2.3 Index test: AT-III activity

AT-III activity was measured using a chromogenic assay on the Sysmex CS-5100 coagulation analyzer and was available within the institutional admission coagulation workflow. The baseline AT-III activity value was defined as the first valid measurement within w0. For temporal analyses, the laboratory information system report release time of this first valid admission AT-III result was extracted as the clinical temporal index.

Admissions without a valid AT-III result within w0 remained in the descriptive cohort but were excluded from AT-III-specific analyses. Representativeness was evaluated by comparing AT-III-available and AT-III-unmeasured admissions with respect to demographic characteristics, clinical severity-group distribution, septic-shock occurrence, and selected non-coagulation laboratory variables (Supplementary Table S3).

The manufacturer reference range was 80–120%. The prespecified threshold of AT-III activity <80% corresponded to the lower reference limit and was used as a clinically interpretable threshold of interest rather than a data-derived cutpoint. AT-III activity <60% was reported as an exploratory severe-deficit stratum for descriptive analyses only. AT-III activity, measured by a functional chromogenic assay, was analytically distinct from AT-III antigen measurement in the exploratory mechanism panel described below.

### 2.4 Reference endpoint and temporal adjudication

The primary endpoint was septic shock, defined according to Sepsis-3 criteria as vasopressor requirement to maintain a mean arterial pressure of at least 65 mmHg after adequate fluid resuscitation, together with lactate >2 mmol/L. (1) The reference endpoint did not include AT-III activity or any raw ISTH score component as a diagnostic criterion. A secondary severe-infection composite endpoint, defined as Sepsis-3 sepsis plus septic shock, was used only for analyses in which primary-endpoint event counts did not support stable estimation.

For temporal adjudication, operational septic-shock onset was defined as the earliest time during the index hospitalization at which both vasopressor treatment and a lactate concentration >2 mmol/L were documented. The report release time of the first valid admission AT-III result was compared with this operational onset time. Septic-shock admissions were classified as: incident shock after AT-III report release; prevalent or concurrent shock at or before AT-III report release; time-indeterminate shock when the available records did not permit reliable temporal ordering; or shock without a valid admission AT-III result.

Temporal adjudication was performed separately from clinical severity classification using recorded AT-III report-release timestamps, vasopressor-treatment records, lactate measurements, and retrospective chart review. Because specimen collection necessarily preceded laboratory report release, AT-III sampling also preceded operational shock onset whenever AT-III report release preceded onset. This analysis reconstructed temporal ordering retrospectively and was not intended as prospective workflow validation.

### 2.5 Comparator frameworks: ISTH 2001 and 2025 SSC overt-DIC scores

The ISTH 2001 overt-DIC score and the 2025 ISTH Scientific and Standardization Committee (SSC) overt-DIC score were calculated using admission-window laboratory data. The ISTH 2001 score was based on platelet count, coagulation-time abnormality, fibrinogen, and D-dimer, with overt DIC defined as a total score of ≥5.

The 2025 ISTH SSC overt-DIC score was calculated from platelet count, D-dimer relative to the assay-specific upper limit of normal, prothrombin-time prolongation, and fibrinogen concentration. Platelet count <50 × 10^9^/l and 50–<100 × 10^9^/l were assigned 2 and 1 points, respectively; D-dimer >3–≤7 times and >7 times the upper limit of normal were assigned 2 and 3 points, respectively; prothrombin-time prolongation of ≥3–<6 s and ≥6 s was assigned 1 and 2 points, respectively; and fibrinogen <1.0 g/L was assigned 1 point. Overt DIC was defined as a total score of ≥5.

D-dimer was reported in ng/ml fibrinogen-equivalent units, with an upper limit of normal of 500 ng/mL. Paired comparisons of AT-III activity, the ISTH 2001 score, and the 2025 ISTH SSC score were performed in patients with complete data for all score components.

### 2.6 Covariates, analytic subsets, and lactate selection

Prespecified covariates entered multivariable clinical models as raw laboratory measurements: age, sex, platelet count, international normalized ratio, fibrinogen, D-dimer, neutrophil-to-lymphocyte ratio, serum creatinine, and serum albumin for the hepatic-function sensitivity model. Sex was included in accordance with SAGER recommendations (23), and sex-specific interaction was assessed in subgroup analyses.

**Figure 1:**
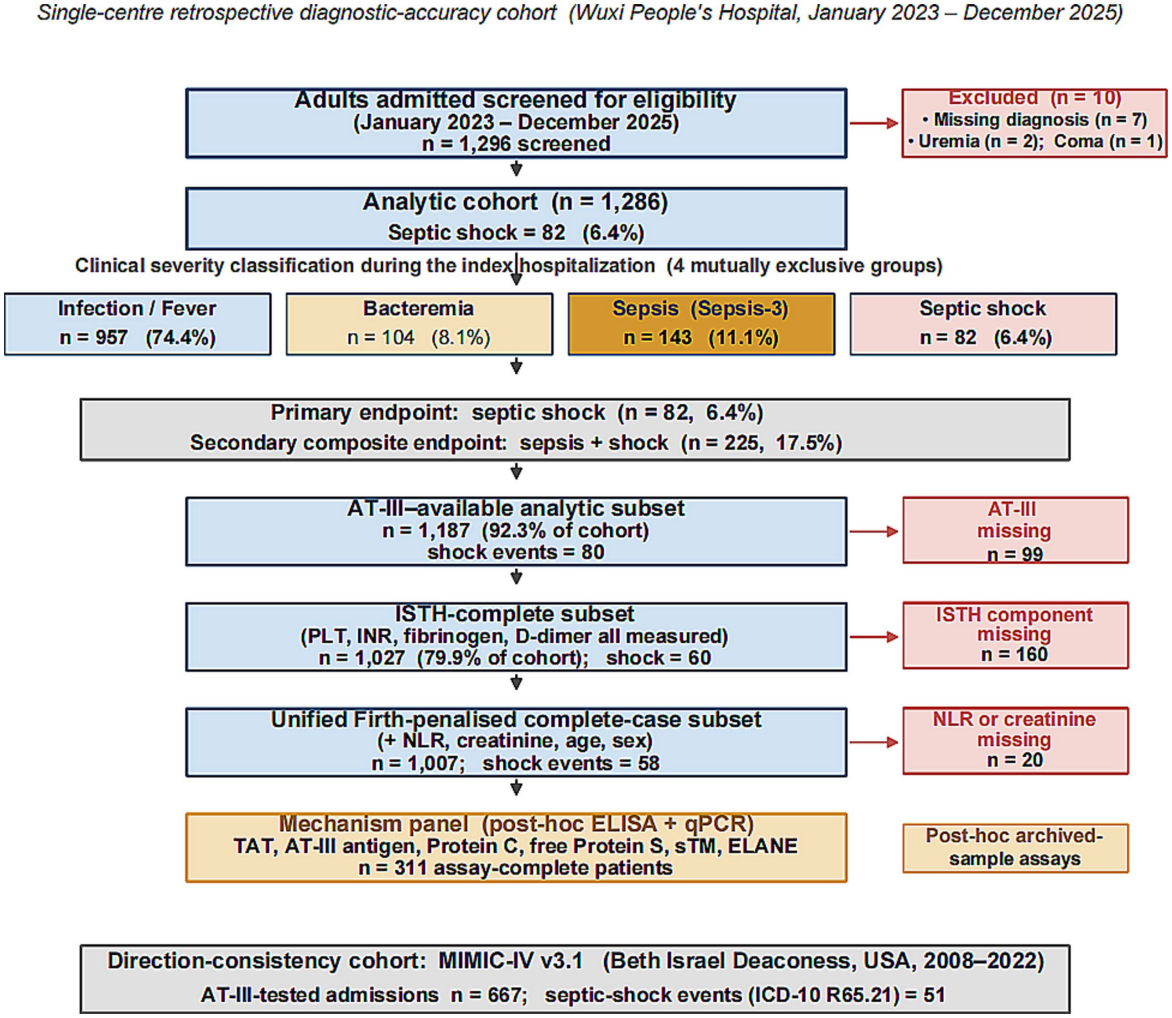
Patient-flow diagram and analysis-specific subsets. Flow of screened admissions through the discovery cohort and analytic subsets. Clinical severity groups represent the highest severity reached during the index hospitalization. The diagram shows the AT-III-available subset, ISTH-complete subset, unified complete-case subset for Firth-penalized models, exploratory mechanism panel, and selected MIMIC-IV AT-III-tested direction-consistency cohort. Detailed subset definitions and event counts are provided in Supplementary Table S1.

Continuous covariates were standardized to per-1-SD increments. AT-III activity was reported in the deficit direction throughout: odds ratios greater than 1 indicate higher odds of septic shock per 1-SD decrease in AT-III activity. Reciprocal estimates were provided where relevant in table and figure footnotes. Representativeness of admissions with versus without a valid admission AT-III result is reported in Supplementary Table S3. Missingness of the four ISTH component variables by clinical-severity stratum is reported in Supplementary Table S26. Analysis-specific complete-case denominators were defined by availability of the variables required for each analysis; subset relationships are shown in Figure 1 and Supplementary Table S1; reconciliation of analysis-specific denominators and septic-shock event counts is provided in Supplementary Table S27.

Lactate testing was clinically indicated rather than uniformly performed. For lactate analyses, values were selected within a prespecified ±24-h window relative to AT-III report release. A hierarchical temporal rule was applied: an eligible lactate measurement obtained before AT-III report release was preferentially selected whenever available; if no eligible pre-report lactate value was available, the closest eligible post-report lactate measurement was selected. No lactate values were imputed.

The primary lactate-analysis subset included admissions with a valid AT-III activity result and a selected lactate measurement. In the incident-shock analysis, incident septic-shock cases were included only when both AT-III report release and the selected lactate measurement preceded operational shock onset; non-shock admissions with the required laboratory data were retained as comparators. Lactate-based analyses were interpreted as pragmatic comparisons with a commonly used bedside marker in a clinically selected subset and not as complete adjustment for global illness severity.

### 2.7 Exploratory mechanism panel

The exploratory mechanism panel comprised 311 assay-complete admissions. Protein C activity, measured by a chromogenic method, and free Protein S activity, measured by a clotting assay, were obtained through routine standardized methods in the institutional clinical coagulation laboratory.

The remaining analytes were measured retrospectively as post-hoc research assays in a single batch on June 30, 2025. Archived citrate plasma had been retained from blood collected on the admission day, although it was not necessarily the first blood draw of the admission. Citrate-plasma AT-III antigen (ELISA; Abcam ab222507), thrombin–antithrombin complex (TAT; ELISA; Abcam ab108907), and soluble thrombomodulin (sTM; ELISA; Abcam ab46508) were measured by sandwich ELISA. ELANE mRNA fold change was assessed by two-step quantitative reverse-transcription polymerase chain reaction using SYBR Green chemistry, with ACTB as the reference gene. Primer sequences, amplification efficiency, melt-curve validation, and assay-quality details are provided in Supplementary methods section S2.7. Run-level quality-control summaries and records for the post-hoc ELISA and qRT-PCR assays are provided in Supplementary Tables S32–S34. De-identified mechanism-panel data are provided in Supplementary Table S35.

AT-III activity was measured in fresh clinical samples, whereas AT-III antigen and related analytes were measured retrospectively in archived admission-day citrate plasma. The activity-to-antigen ratio therefore paired values at the patient level but did not require prospectively matched blood draws or identical pre-analytical handling. Complete freeze–thaw histories before post-hoc batch testing were not uniformly recoverable from source records.

The mechanism subset included admissions enrolled before the final batch-processing date with complete measurements required for the exploratory panel. All contributing ELISA and quantitative reverse-transcription polymerase chain reaction runs met the documented run-level quality-control criteria; this designation reflected analytical-run quality control and not patient-level exclusion by a Westgard rule. Storage-duration sensitivity analyses were performed to examine whether the exploratory activity-to-antigen pattern was materially altered after accounting for storage duration. The mechanism panel was prespecified as exploratory biological-plausibility analysis rather than a primary diagnostic-accuracy comparator.

### 2.8 MIMIC-IV cross-cohort direction-consistency analysis

This study identified MIMIC-IV v3.1 admissions with at least one AT-III activity measurement (itemid 51,140) through the PhysioNet credentialed-user pathway under a signed Data Use Agreement. AT-III values outside a plausibility range of 10–200% were excluded. Because AT-III testing in MIMIC-IV was selectively ordered, this cohort was not considered representative of all intensive-care admissions with infection or sepsis.

In MIMIC-IV, septic shock was operationalized using International Classification of Diseases, Tenth Revision code R65.21 because comprehensive Sepsis-3 ascertainment was not feasible across the archived dataset. The cross-cohort analysis used a minimal portable model including age, sex, and AT-III activity; mechanism-panel analytes were unavailable. This analysis assessed the direction consistency of the AT-III association in a selected AT-III-tested cohort and was not an external validation of the discovery-cohort clinical models.

### 2.9 Statistical analysis

Continuous variables are summarized as median [interquartile range] and compared across clinical severity groups using the Kruskal–Wallis test with Jonckheere–Terpstra trend testing. Categorical variables are compared using the chi-square test or Fisher’s exact test, as appropriate. Discrimination was quantified using the area under the receiver-operating-characteristic curve (AUC) with 2,000-replicate bootstrap percentile 95% confidence intervals. Proportion-based 95% confidence intervals were calculated using the Wilson method (24). Pairwise AUC comparisons used the DeLong method (25). Comparisons of AT-III activity, ISTH 2001, and ISTH 2025 SSC scores were performed within the ISTH-complete subset to satisfy paired-comparison assumptions. Cohen’s kappa quantified agreement between the two ISTH frameworks. Youden-optimal cutpoints were reported as contextual summaries and did not replace the prespecified AT-III < 80% threshold.

The primary clinical regression model, Model 1b, was a Firth bias-reduced logistic regression model of septic shock including age, sex, platelet count, international normalized ratio, fibrinogen, D-dimer, and AT-III activity. The nested base model, Model 1a, included age, sex, and the raw ISTH components without AT-III. Model 2 added neutrophil-to-lymphocyte ratio and serum creatinine to Model 1b. These models were fit on the same unified complete-case subset to permit like-with-like paired AUC comparison. A hepatic-function sensitivity model, Model 2b, additionally included serum albumin. The final models had a modest number of events per candidate covariate—approximately 8.3 for Model 1b (58 septic-shock events, seven covariates) and 6.4 for Model 2 (58 events, nine covariates)—and the primary below-threshold analysis included 44 septic-shock events (26). For Model 1b, apparent discrimination, calibration slope, Brier score, and Hosmer–Lemeshow goodness-of-fit were reported. Internal optimism was estimated using 500 Harrell–Steyerberg bootstrap iterations (27). Given the limited event counts, Firth penalization, bootstrap optimism correction, and calibration assessment were used to mitigate small-sample bias and overfitting.

Temporal analyses described the ordering of AT-III report release and operational shock onset. Lactate-adjusted analyses used Firth logistic regression models including age, sex, lactate, and AT-III activity in the clinically indicated lactate-measured subset and in the temporally adjudicated incident-shock analysis. Discrimination of models with and without AT-III was compared using paired AUC methods within the corresponding analytic subset.

Additional supportive analyses used least absolute shrinkage and selection operator (LASSO)-based variable selection and multiple imputation by chained equations (MICE) for missing-data sensitivity analysis; their detailed specifications are provided below (28, 29). Category-free net reclassification improvement and integrated discrimination improvement were used to assess the incremental contribution of AT-III activity beyond raw ISTH-component models (30). The four-component base model included platelet count, international normalized ratio, fibrinogen, and D-dimer; the augmented model additionally included AT-III activity. These analyses did not include demographic covariates. Primary-endpoint reclassification estimates were interpreted as exploratory because of the limited number of septic-shock events and the known interpretive limitations of reclassification metrics; secondary severe-infection composite analyses were considered supportive (31).

Decision-curve analyses also used raw coagulation-component models without demographic covariates (32). For the primary septic-shock endpoint within the ISTH 2001 below-threshold subset, the base model comprised platelet count, international normalized ratio, fibrinogen, and D-dimer, and the augmented model additionally included AT-III activity. Secondary severe-infection composite decision-curve analyses compared the four-component ISTH model with the corresponding AT-III-augmented model. Decision curves were displayed across threshold probabilities from 0 to 50%, with interpretation focused on clinically plausible thresholds of 5–30%. All decision-analytic analyses were interpreted as exploratory. The overall approach to regression-model specification, calibration, internal validation, and cautious interpretation of model performance followed established prediction-modelling principles (33).

Each analysis used the smallest complete-case subset compatible with its variable requirements. AT-III-available and AT-III-unmeasured admissions were compared to assess potential selection associated with test availability. MICE was performed only as a sensitivity analysis for the secondary severe-infection composite endpoint and did not define the primary estimates.

Prespecified subgroup analyses assessed AT-III associations across seven strata using interaction terms. Internal70/30 split-sample replication, restricted cubic spline dose–response analysis, LASSO modelling, Bayesian posterior estimation of the exploratory AT-III-plus-TAT two-marker mechanism-panel model, and the SIC-stratified partial-SOFA analysis were considered supportive or exploratory analyses. Because SIC includes organ-dysfunction components, the SIC-stratified analysis was interpreted as supportive rather than independent evidence. Two-sided *p* values <0.05 were used for primary analyses. Subgroup, sensitivity, and exploratory analyses were interpreted descriptively without multiplicity-adjusted confirmatory inference. All analyses were performed using Python 3.12 (pandas 2.2, scikit-learn 1.4, statsmodels 0.14, and PyMC 5.15) and R version 4.5.1 (logistf 1.26 and pROC 1.18).

## 3 Results

### 3.1 Cohort characteristics and AT-III severity gradient

A total of 1,286 adults admitted with infection between January 2023 and December 2025 met eligibility criteria [median age, 51 years (IQR, 43–60); 588 (45.7%) male] (Figure 1; Table 1; Supplementary Table S1). Clinical severity classification for the index hospitalization assigned 957 patients (74.4%) to the infection/fever group, 104 (8.1%) to bacteremia without Sepsis-3 criteria, 143 (11.1%) to Sepsis-3 sepsis, and 82 (6.4%) to septic shock.

**Table 1:** Characteristics of the study population by highest clinical severity reached during the index hospitalization.

Characteristic	Overall (*n* = 1,286)	Infection/Fever (*n* = 957)	Bacteremia (*n* = 104)	Sepsis (*n* = 143)	Septic shock (*n* = 82)	*p* value
Age, years, median [IQR]	51.0 [43.0–60.0]	51.0 [43.0–60.0]	51.0 [43.8–59.0]	52.0 [43.0–63.0]	50.0 [43.5–60.0]	0.741
Sex, male, *n* (%)	588 (45.7%)	426 (44.5%)	46 (44.2%)	74 (51.7%)	42 (51.2%)	0.290
AT-III activity (%)	80.0 [67.0–92.0]	83.0 [71.0–96.0]	79.0 [63.0–93.0]	70.0 [56.0–82.0]	56.8 [45.8–68.2]	<0.001
Platelet count (×10^9^/l)	184.0 [124.2–256.0]	195.0 [140.0–262.0]	131.0 [39.0–226.5]	153.0 [81.0–230.0]	124.0 [74.0–204.0]	<0.001
INR	1.1 [1.1–1.2]	1.1 [1.1–1.2]	1.1 [1.1–1.2]	1.2 [1.1–1.3]	1.3 [1.1–1.5]	<0.001
Fibrinogen (g/l)	4.1 [3.3–5.0]	4.0 [3.3–4.9]	4.3 [3.2–4.9]	4.5 [3.6–5.4]	4.0 [3.1–4.9]	0.012
D-dimer (ng/ml FEU)	442.0 [230.5–916.5]	380.0 [201.5–720.8]	474.0 [278.5–894.5]	892.5 [447.8–1916.8]	2398.0 [1041.5–6336.5]	<0.001
NLR	4.0 [2.0–8.1]	3.4 [1.9–6.5]	2.3 [0.6–6.9]	8.8 [4.7–18.9]	10.0 [5.6–17.9]	<0.001
CRP (mg/l)	75.2 [27.6–166.4]	47.0 [14.5–121.6]	87.8 [27.7–162.2]	126.2 [53.2–199.8]	147.3 [73.5–203.9]	<0.001
Albumin (g/l)	32.6 [28.9–36.0]	33.5 [29.9–36.7]	30.7 [27.4–34.1]	28.6 [25.5–33.3]	28.1 [25.7–31.1]	<0.001
Creatinine (μmol/l)	69.4 [56.4–91.9]	68.3 [56.0–84.9]	66.3 [54.3–86.7]	80.0 [60.1–152.2]	97.8 [64.7–138.2]	<0.001
Total bilirubin (μmol/l)	10.6 [8.1–14.7]	10.4 [7.9–13.8]	10.8 [8.3–15.9]	11.8 [8.3–16.0]	15.2 [9.6–18.6]	<0.001
ALT (U/l)	22.0 [13.1–41.1]	21.9 [13.1–39.7]	20.8 [13.1–41.0]	20.5 [11.0–42.3]	31.2 [19.1–49.1]	0.186
AST (U/l)	24.0 [17.0–41.0]	24.0 [17.0–39.0]	24.0 [17.0–42.0]	23.0 [17.0–44.0]	38.0 [24.0–68.0]	<0.001
LDH (U/l)	210.0 [170.8–269.2]	205.0 [169.0–262.0]	198.0 [168.0–264.0]	220.0 [187.0–295.0]	263.0 [192.0–532.0]	<0.001

Values are median (IQR) for continuous variables and *n* (%) for categorical variables. *p* values by Kruskal–Wallis test (continuous) or *χ*^2^ test (categorical). Severity strata defined per Sepsis-3 criteria. AT-III activity was summarized among admissions with a valid admission-window AT-III result (overall *n* = 1,187; infection/fever *n* = 875; bacteremia *n* = 97; sepsis *n* = 135; septic shock *n* = 80).

**Figure 2:**
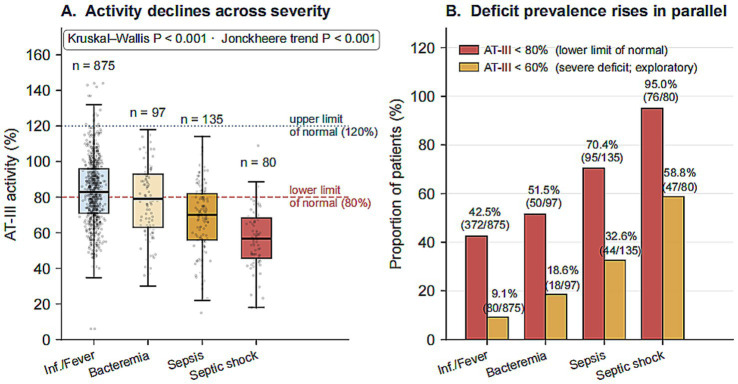
AT-III activity by infection severity. **(A)** AT-III activity across the four clinical severity strata in the AT-III-available subset (*n* = 1,187). Boxplots show median and interquartile range; points represent individual admissions. Horizontal dashed lines indicate the institutional reference range of 80–120%. **(B)** Prevalence of AT-III activity <80 and <60% across severity strata. Trend statistics are reported in Table 1.

A valid admission-window AT-III activity result was available for 1,187 admissions (92.3%), including 80 septic-shock events. Within this AT-III-available subset, AT-III activity declined across the severity spectrum: median activity was 83.0% (IQR, 71.0–96.0) in infection/fever, 79.0% (63.0–93.0) in bacteremia, 70.0% (56.0–82.0) in sepsis, and 56.8% (45.8–68.2) in septic shock (Kruskal–Wallis *p* < 0.001; Jonckheere–Terpstra trend *p* < 0.001; Figure 2; Table 1). The prevalence of AT-III activity <80% increased from 42.5% (372/875) in infection/fever to 95.0% (76/80) in septic shock, whereas the prevalence of AT-III activity <60% increased from 9.1% (80/875) to 58.8% (47/80), respectively.

Admissions with and without a valid admission AT-III result did not show statistically clear differences in age, sex, overall clinical severity distribution, creatinine, and C-reactive protein; albumin differed modestly between groups (Supplementary Table S3). Septic shock occurred in 80 of 1,187 AT-III-available admissions (6.7%) and in 2 of 99 AT-III-unmeasured admissions (2.0%; *p* = 0.083). In the full AT-III-available subset, AT-III activity alone discriminated septic shock with an AUC of 0.834 (95% CI, 0.790–0.872; Table 2).

**Table 2:** Diagnostic performance of AT-III activity for septic shock and the severe-infection composite endpoint.

Endpoint	*n*	Events	AUC	AUC 95% CI	Best cutoff	Sensitivity	Specificity	PPV	NPV
Primary	1,187	80	0.834	0.790–0.872	69.0	0.750	0.755	0.181	0.977
Secondary	1,187	215	0.760	0.724–0.794	72.0	0.633	0.733	0.343	0.900

Primary endpoint: septic shock according to Sepsis-3 criteria. Secondary endpoint: severe-infection composite, defined as Sepsis-3 sepsis plus septic shock. “Best cutoff” denotes the Youden-optimal value and is reported for contextual interpretation only; the prespecified clinical threshold remains AT-III activity <80%. AUC, area under the receiver operating characteristic curve; CI, confidence interval; PPV, positive predictive value; NPV, negative predictive value. Estimates are based on the AT-III–available subset (*n* = 1,187).

### 3.2 AT-III stratification within the ISTH below-threshold stratum

**Figure 3:**
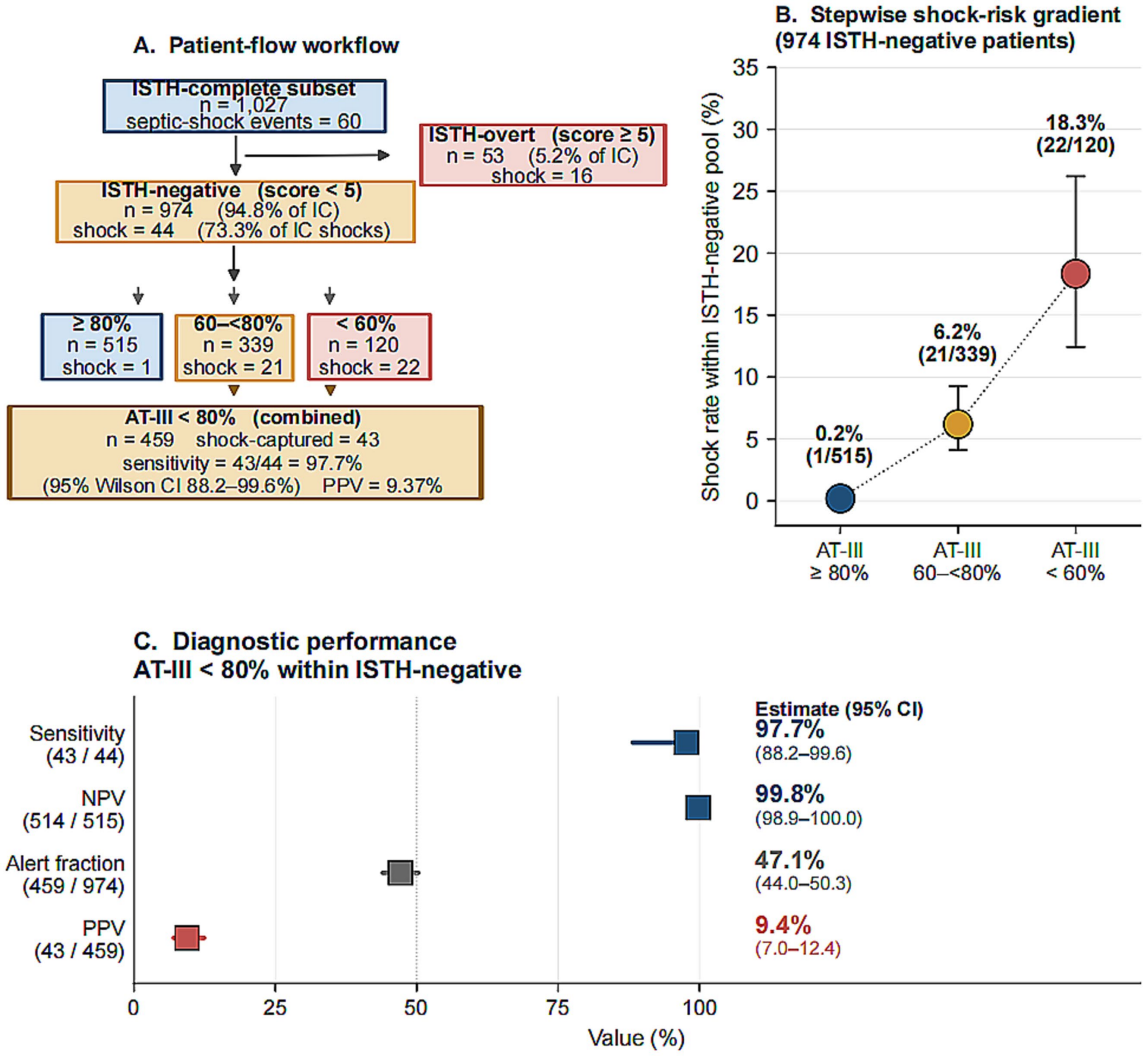
AT-III risk enrichment within the ISTH below-threshold subgroup. **(A)** Distribution of septic-shock events across the ISTH-complete subset and prespecified AT-III categories within the ISTH 2001 below-threshold subgroup (score <5). **(B)** Septic-shock rates across AT-III categories within the below-threshold subgroup; point estimates are shown with Wilson 95% confidence intervals. **(C)** Diagnostic characteristics of the prespecified AT-III < 80% threshold within the below-threshold subgroup. Supporting data are provided in Supplementary Table S5.

Among 1,027 patients with all four ISTH scoring components available within the admission window, 974 (94.8%) were below the ISTH 2001 overt-DIC threshold (score <5), whereas 53 (5.2%) met the overt-DIC threshold. Of the 60 septic-shock events in this ISTH-complete subset, 44 (73.3%) occurred in the below-threshold stratum and 16 occurred in the overt-DIC stratum (Figure 3A; Table 3). Because the below-threshold stratum contained most component-complete admissions, the predominance of septic-shock events in this stratum partly reflected its size.

**Table 3:** Distribution of ISTH DIC scores and overt DIC by severity group.

Score framework	Severity group	*n*	Median	Q1 (25th)	Q3 (75th)	Overt DIC (n)	Overt DIC (%)
ISTH 2001 (complete)	Infection/Fever	772	1	0	2	9	1.2
ISTH 2001 (complete)	Bacteremia	86	2	1	3	12	14.0
ISTH 2001 (complete)	Sepsis	109	3	1	4	16	14.7
ISTH 2001 (complete)	Septic shock	60	4	3	5	16	26.7
ISTH 2025 SSC (overt-DIC)	Infection/Fever	772	0	0	1	4	0.5
ISTH 2025 SSC (overt-DIC)	Bacteremia	86	1	0	2	4	4.7
ISTH 2025 SSC (overt-DIC)	Sepsis	109	1	0	2	7	6.4
ISTH 2025 SSC (overt-DIC)	Septic shock	60	3	1	4	10	16.7

Overt DIC was defined as a total score of ≥5 under both frameworks. The 2025 ISTH SSC score used platelet count, D-dimer relative to the assay-specific upper limit of normal, prothrombin-time prolongation, and fibrinogen concentration. Both scores and paired AUC comparisons used the same admission-window component-complete subset (*n* = 1,027; 60 septic-shock events). ISTH, International Society on Thrombosis and Haemostasis; SSC, Scientific and Standardization Committee; DIC, disseminated intravascular coagulation.

Within the ISTH below-threshold stratum, AT-III activity discriminated septic shock with an AUC of 0.844 (95% CI, 0.801–0.887), similar to the AUC across the full ISTH-complete subset (0.850; 95% CI, 0.808–0.888). Septic-shock frequency increased stepwise across prespecified AT-III categories: 0.2% (1/515) among patients with AT-III activity ≥80, 6.2% (21/339) among those with activity of 60 to <80%, and 18.3% (22/120) among those with activity <60% (Figure 3B; Supplementary Table S5).

Among the 974 below-threshold admissions, 459 (47.1%) had AT-III activity <80%. Of the 44 septic-shock cases within the below-threshold stratum, 43 had AT-III activity <80% (97.7%; 95% Wilson CI, 88.2–99.6%) and 22 had activity <60% (50.0%; Figure 3; Supplementary Table S5). The positive predictive value of AT-III activity <80% in this stratum was 9.37% (43/459), corresponding to a number needed to flag of approximately 11 admissions to identify one septic-shock case. Conversely, 416 of 459 below-threshold admissions with AT-III activity <80% did not have septic shock, indicating that the prespecified threshold provided high-sensitivity risk enrichment rather than rule-in diagnosis.

### 3.3 Retrospectively reconstructed temporal ordering and selected lactate analyses

**Figure 4:**
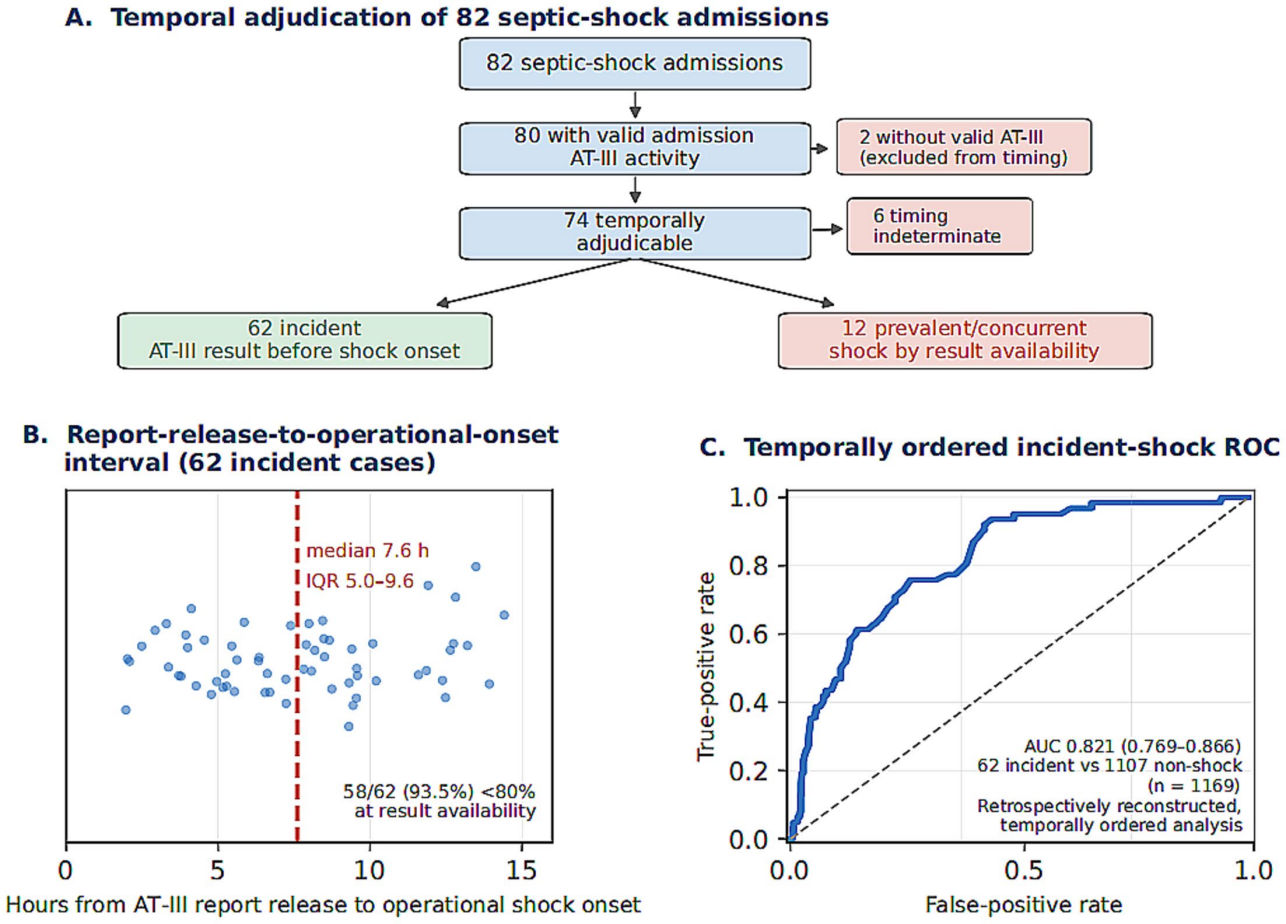
Temporal ordering of AT-III results availability and septic-shock onset. **(A)** Temporal adjudication among 82 septic-shock admissions. Of 80 admissions with valid admission AT-III activity, 62 had incident shock after AT-III result availability, 12 had prevalent or concurrent shock, and 6 had indeterminate temporal ordering. **(B)** Interval from first valid AT-III report release to operational shock onset among the 62 incident cases. **(C)** Receiver-operating-characteristic curve for AT-III activity in 62 incident septic-shock cases versus 1,107 non-shock admissions. Operational shock onset was defined as the earliest time at which vasopressor treatment and lactate >2 mmol/L were both documented. Additional temporal-adjudication details and de-identified temporal/lactate source data are provided in Supplementary Tables S28, S29.

Among 82 septic-shock admissions, 80 had a valid admission AT-III result. Of these 80 admissions, 74 were temporally adjudicable: 62 were classified as incident shock after AT-III report release and 12 as prevalent or concurrent shock at or before AT-III report release. Six additional admissions had indeterminate temporal ordering, and two septic-shock admissions had no valid admission AT-III result and were not included in AT-III-specific temporal analyses (Figure 4; Supplementary Table S28).

Among the 62 incident cases, first valid AT-III report release preceded operational septic-shock onset by a median of 7.6 h (IQR, 5.0–9.6 h). Because blood collection necessarily preceded report release, AT-III sampling also preceded operational shock onset in these cases. At AT-III result availability, 58 of 62 incident cases (93.5%) had AT-III activity <80%. In the retrospectively adjudicated temporal analysis comparing 62 incident septic-shock cases with 1,107 non-shock admissions, AT-III activity yielded an AUC of 0.821 (95% CI, 0.769–0.866; Figure 4; Supplementary Table S28). This retrospective ordering supports a short-horizon temporal association but does not establish prospective workflow performance.

Lactate was measured on clinical indication in 520 admissions. Of these, 518 had both a valid admission AT-III result and a selected lactate measurement within the prespecified time window and comprised the primary lactate-analysis subset, including 80 septic-shock events. In this selected subset, AT-III activity alone had an AUC of 0.805 and lactate alone had an AUC of 0.745. In models including age, sex, and lactate, addition of AT-III activity increased the AUC from 0.740 to 0.821 (incremental ΔAUC, 0.081; *p* = 0.001). Lower AT-III activity remained conditionally associated with septic shock after adjustment for age, sex, and lactate (OR, 2.87 per 1-SD decrease; 95% CI, 2.09–3.96; likelihood-ratio *p <* 0.001; Supplementary Table S30).

In the temporally ordered incident-shock analysis, 500 admissions with the required laboratory data were included, comprising 62 incident septic-shock events. In all 62 incident cases, both AT-III report release and the selected lactate measurement preceded operational shock onset. Adding AT-III activity to age, sex, and lactate increased the AUC from 0.680 to 0.792 (incremental ΔAUC, 0.112; *p* = 0.002), and the lactate-adjusted AT-III association remained evident (OR, 2.88 per 1-SD decrease; 95% CI, 2.06–4.03; likelihood-ratio *p* < 0.001; Supplementary Table S30). Because lactate testing was clinically indicated rather than uniformly performed and lactate contributes to the operational septic-shock definition, these analyses were interpreted as selected-subset comparisons with a commonly used bedside marker rather than complete adjustment for global illness severity.

### 3.4 Contextual ISTH comparisons, conditional clinical models, and incremental analyses

**Figure 5:**
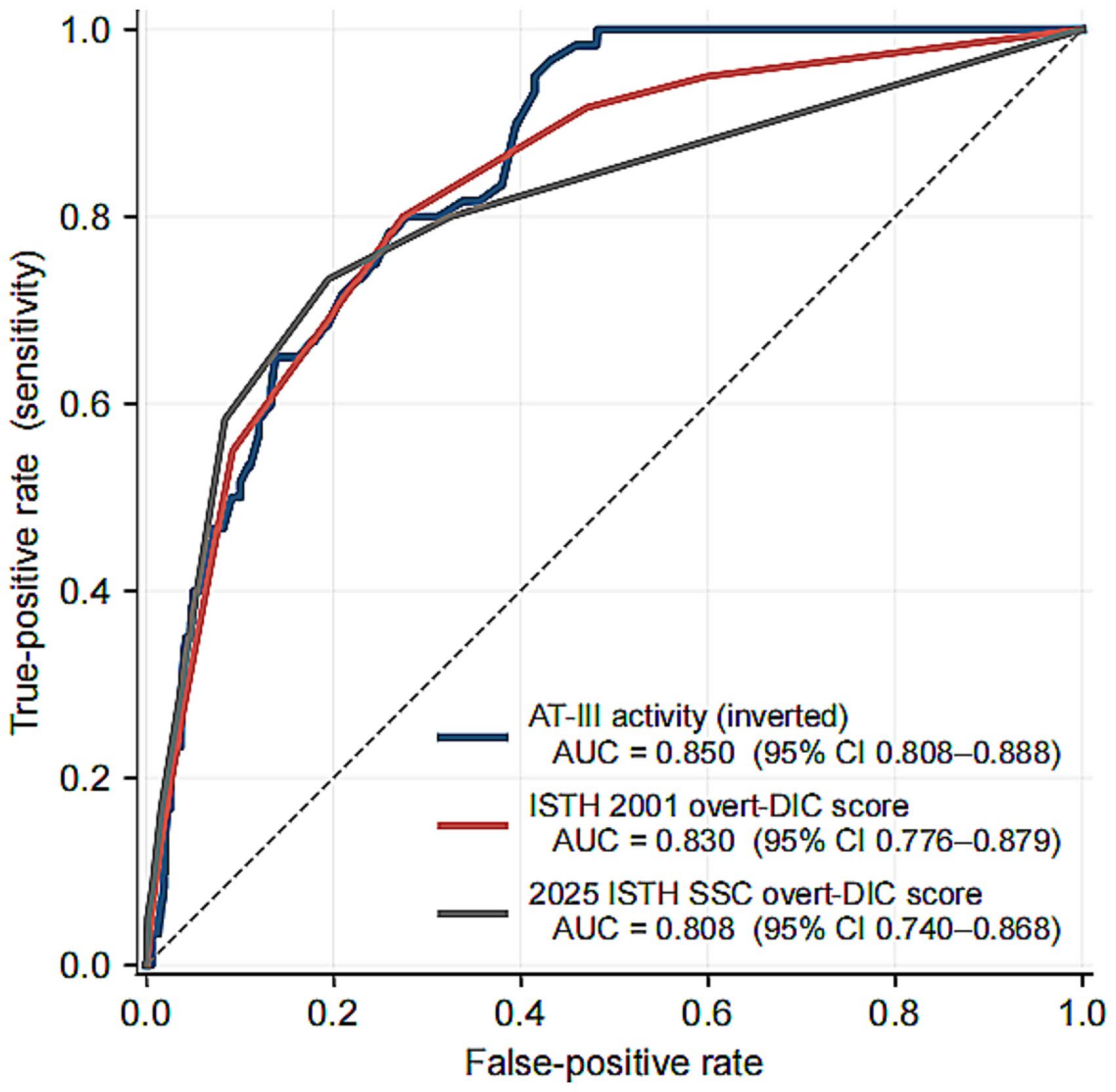
Septic-shock discrimination in the ISTH-complete subset. Receiver-operating-characteristic curves for AT-III activity, the ISTH 2001 overt-DIC score, and the 2025 ISTH SSC overt-DIC score in the shared score-complete subset (*n* = 1,027; 60 septic-shock events). AT-III activity was inverted so that lower activity corresponded to higher estimated septic-shock risk. Paired comparisons are reported in Supplementary Table S4. These comparisons are contextual for the septic-shock endpoint and do not evaluate DIC-classification performance.

Comparisons with ISTH frameworks were used to contextualize AT-III discrimination for the septic-shock endpoint and were not intended to assess the performance of either framework for its primary purpose of overt-DIC classification. Within the common ISTH-complete subset, AUCs for septic-shock discrimination were 0.850 (95% CI, 0.808–0.888) for AT-III activity, 0.830 (95% CI, 0.776–0.879) for the ISTH 2001 score, and 0.808 (95% CI, 0.740–0.868) for the 2025 ISTH SSC score (Figure 5; Supplementary Table S4). The paired comparisons of AT-III activity with the ISTH 2001 score (DeLong *p* = 0.49) and with the 2025 SSC score (*p* = 0.23) were not statistically significant. The two ISTH frameworks showed substantial agreement for overt-DIC classification (Cohen’s *κ* = 0.629); overt-DIC prevalence was 5.2% (53/1,027) under the 2001 framework and 2.4% (25/1,027) under the 2025 SSC score (Table 3; Supplementary Table S4). Among individual coagulation markers in the AT-III–available subset, AT-III activity discriminated septic shock comparably to D-dimer (DeLong *p* = 0.88) and significantly better than INR, prothrombin time, activated partial thromboplastin time, fibrinogen, and platelet count (all *p* < 0.001); single-marker discrimination and paired comparisons across both endpoints are summarized in Supplementary Tables S22, S23.

**Figure 6:**
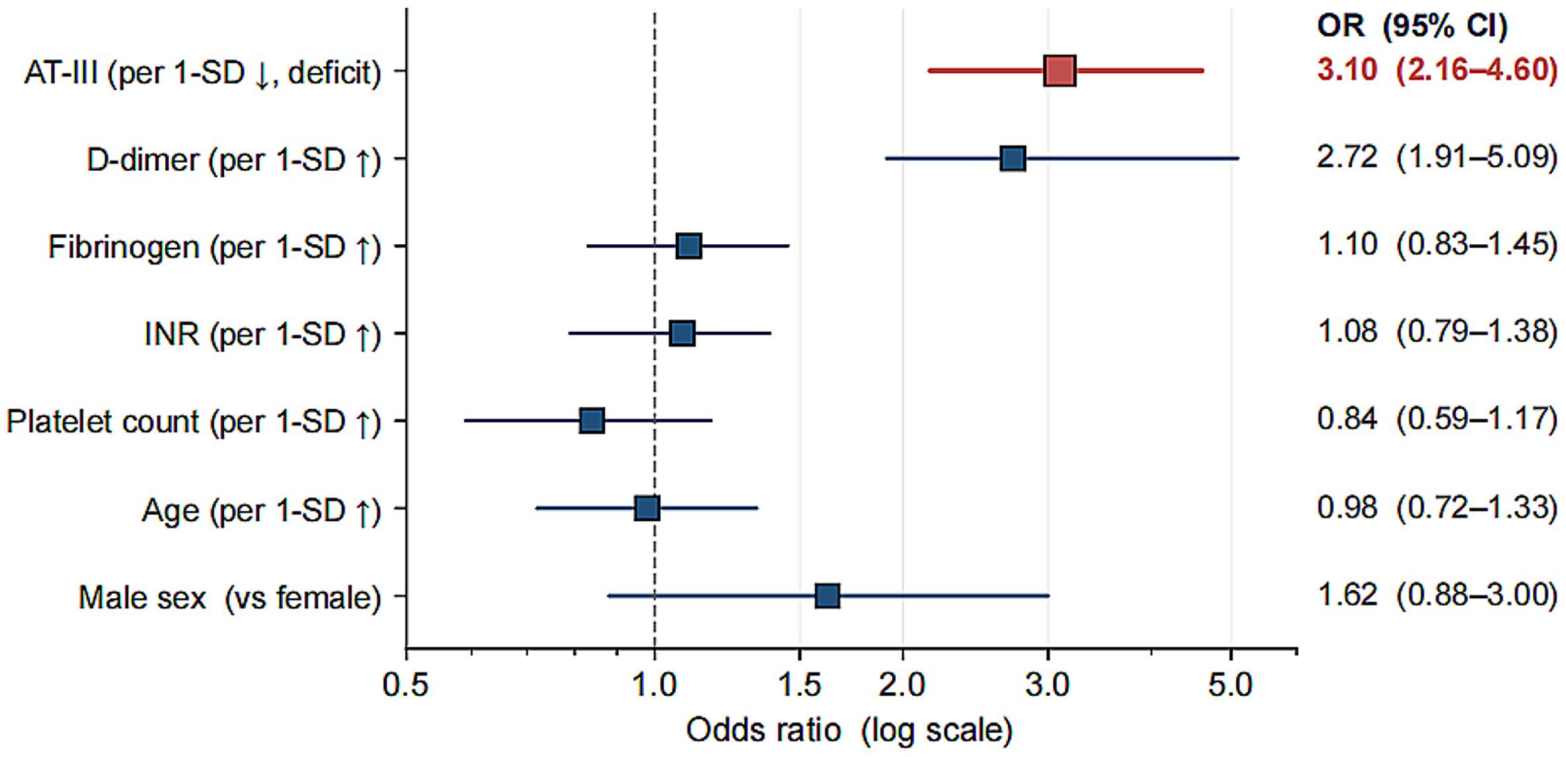
Firth-penalized Model 1b for septic shock. Forest plot of odds ratios from Model 1b in the unified complete-case subset (*n* = 1,007; 58 septic-shock events). Model 1b included age, sex, platelet count, INR, fibrinogen, D-dimer, and AT-III activity. Non-AT-III continuous variables are expressed per 1-standard-deviation increase. AT-III activity is shown in the deficit direction, per 1-standard-deviation decrease. Full model results are reported in Table 4 and Supplementary Tables S14, S16.

In the unified complete-case subset of 1,007 admissions with 58 septic-shock events, Firth-penalized logistic regression showed a conditional association between lower AT-III activity and septic shock after adjustment for age, sex, platelet count, INR, fibrinogen, and D-dimer. Model 1a, comprising age, sex, and raw ISTH components, had an apparent AUC of 0.834 (95% CI, 0.772–0.890). Adding AT-III activity in Model 1b increased the apparent AUC to 0.900 (95% CI, 0.872–0.928; paired DeLong *p* = 0.006). The AT-III coefficient corresponded to an OR of 3.10 per 1-SD decrease in activity (95% CI, 2.16–4.60; *p* < 0.001; Table 4; Figure 6; Supplementary Tables S14, S16).

**Table 4:** Multivariable Firth-penalized logistic regression for septic shock.

Model	Characteristic	OR per 1-SD	95% CI low	95% CI high	*P* value	SD scaling	*n*	Events	AUC
Model 1a	Age	0.996	0.743	1.333	0.977	9.99	1,007	58	0.834
Model 1a	Male sex	1.519	0.851	2.744	0.161	-	1,007	58	0.834
Model 1a	Platelet	0.577	0.403	0.807	0.002	106.83	1,007	58	0.834
Model 1a	INR	1.442	1.173	1.757	<0.001	0.268	1,007	58	0.834
Model 1a	Fibrinogen	1.112	0.842	1.458	0.448	1.271	1,007	58	0.834
Model 1a	D-dimer	3.347	1.790	10.986	<0.001	4887.66	1,007	58	0.834
Model 1b	Age	0.981	0.724	1.327	0.902	9.990	1,007	58	0.900
Model 1b	Male sex	1.619	0.884	3.002	0.123	—	1,007	58	0.900
Model 1b	Platelet	0.838	0.586	1.168	0.315	106.830	1,007	58	0.900
Model 1b	INR	1.085	0.788	1.382	0.570	0.268	1,007	58	0.900
Model 1b	Fibrinogen	1.102	0.830	1.449	0.495	1.271	1,007	58	0.900
Model 1b	D-dimer	2.718	1.907	5.091	<0.001	4887.66	1,007	58	0.900
Model 1b	AT-III deficit (per 1-SD decrease in activity)	3.101	2.155	4.596	<0.001	19.470	1,007	58	0.900
Model 2	Age	0.968	0.712	1.312	0.835	9.99	1,007	58	0.905
Model 2	Male sex	1.577	0.859	2.932	0.146	-	1,007	58	0.905
Model 2	Platelet	0.866	0.604	1.209	0.415	106.83	1,007	58	0.905
Model 2	INR	0.969	0.672	1.342	0.859	0.27	1,007	58	0.905
Model 2	Fibrinogen	1.093	0.810	1.460	0.555	1.27	1,007	58	0.905
Model 2	D-dimer	2.636	1.859	4.502	<0.001	4887.66	1,007	58	0.905
Model 2	AT-III deficit (per 1-SD decrease)	3.228	2.189	4.936	<0.001	19.47	1,007	58	0.905
Model 2	NLR	1.223	1.002	1.489	0.046	9.55	1,007	58	0.905
Model 2	Creatinine	0.801	0.519	1.057	0.222	94.47	1,007	58	0.905

Primary endpoint: septic shock (Sepsis-3 criteria); *n* = 58 events / *n* = 1,007 unified Firth complete-case subset. Odds ratios per 1-SD increment of each variable (95% CI); AT-III shown in the deficit direction (per 1-SD decrease). Confidence intervals are Firth profile-likelihood, except for Model 1a D-dimer, for which a 500-iteration bootstrap percentile interval was used owing to boundary instability. Internal-validation, hepatic-function, and split-sample results are in the Supplementary materials.

**Figure 7:**
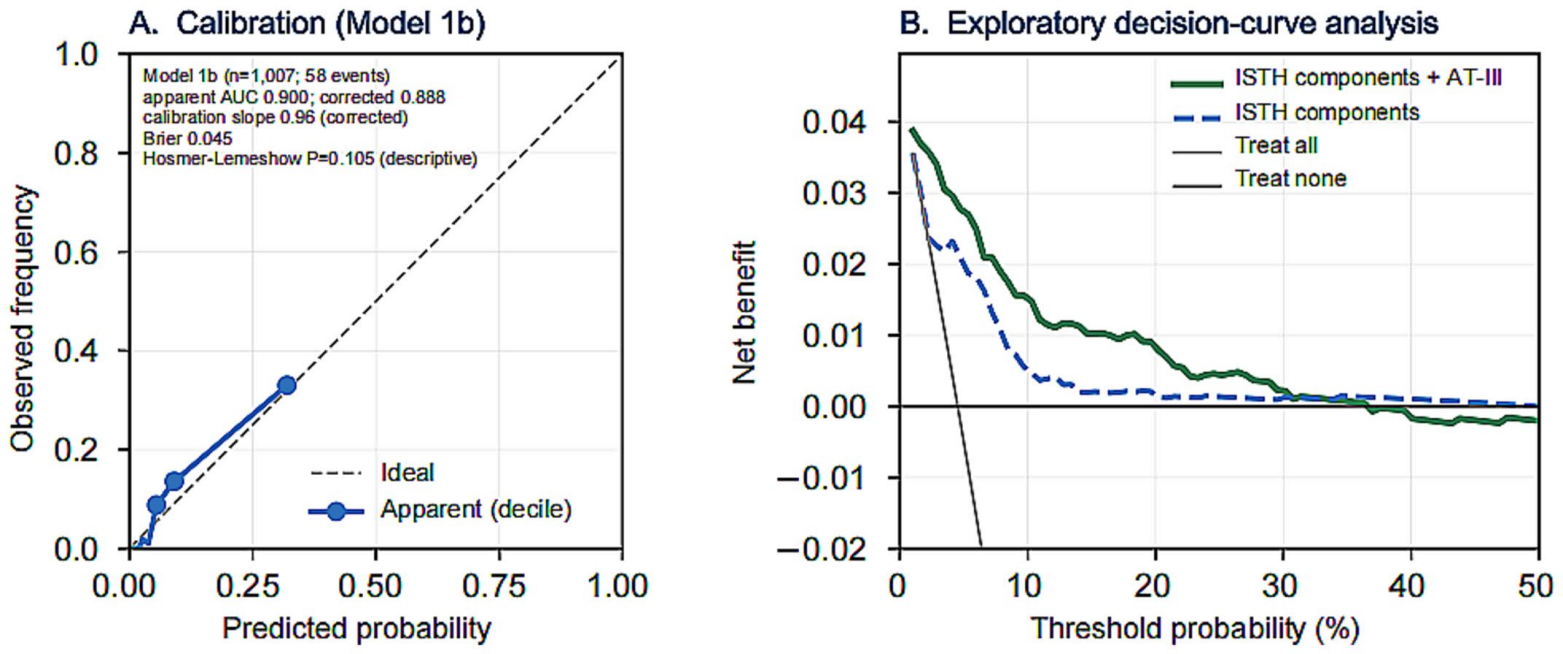
Model 1b calibration and ISTH-component decision-curve analysis. **(A)** Internal calibration assessment of Model 1b in the unified complete-case subset (*n* = 1,007; 58 septic-shock events). Apparent and bootstrap-corrected AUCs, calibration slope, Brier score, and Hosmer–Lemeshow *p* value are shown. **(B)** Exploratory decision-curve analysis for septic shock within the ISTH 2001 below-threshold subgroup (*n* = 974; 44 septic-shock events). The base model included platelet count, INR, fibrinogen, and D-dimer; the augmented model additionally included AT-III activity. Treat-all and treat-none strategies are shown as reference curves.

For Model 1b, bootstrap optimism was 0.012 and the bootstrap-corrected AUC was 0.888. The bootstrap-corrected calibration slope was 0.96, the Brier score was 0.045, and the Hosmer–Lemeshow goodness-of-fit *p* value was 0.105 (Figure 7A; Supplementary Table S7). Model 2, which additionally included neutrophil-to-lymphocyte ratio and creatinine, had an AUC of 0.905 (95% CI, 0.878–0.933) and did not significantly improve discrimination over Model 1b (paired DeLong *p* = 0.25; Supplementary Table S14). Bootstrap optimism-corrected sensitivity and specificity at the Youden-optimal AT-III cutpoint (<69.0%) are provided in Supplementary Table S10.

Separate reclassification analyses used raw ISTH components without demographic covariates as the base model. In the ISTH-complete subset, adding AT-III activity increased AUC for the primary septic-shock endpoint from 0.847 to 0.903 (ΔAUC, 0.056; *p* < 0.001). Category-free net reclassification improvement was 0.688 (95% CI, 0.436–0.935; *p* < 0.001), and integrated discrimination improvement was 0.066 (95% CI, 0.037–0.096; *p* < 0.001). For the secondary severe-infection composite endpoint, AUC increased from 0.758 to 0.812 (ΔAUC, 0.054; *p* < 0.001), with a net reclassification improvement of 0.598 (95% CI, 0.436–0.760; *p* < 0.001) and an integrated discrimination improvement of 0.078 (95% CI, 0.058–0.099; *p* < 0.001; Supplementary Table S12).

In the ISTH below-threshold subset, exploratory decision-curve analysis compared a raw ISTH-component model with the corresponding AT-III-augmented model. Addition of AT-III showed higher net benefit across threshold probabilities of approximately 1–33% (Figure 7B). Decision-curve analyses for the secondary severe-infection composite endpoint are shown in Supplementary Figure S2. Given the retrospective design, the limited number of septic-shock events in this subgroup, and the low positive predictive value of the <80% threshold, this finding was interpreted as exploratory decision-analytic support for risk enrichment rather than evidence for an AT-III-triggered escalation strategy.

### 3.5 Exploratory activity-to-antigen pattern

**Figure 8:**
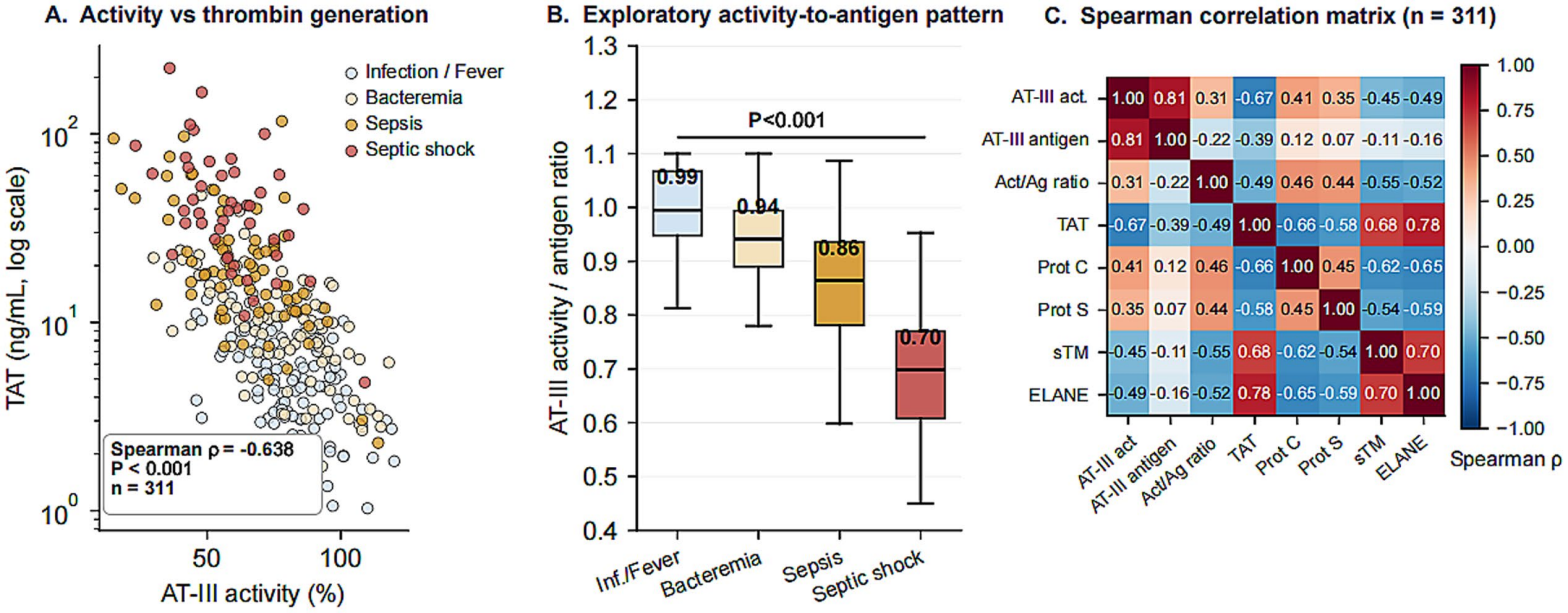
Exploratory activity-to-antigen and ancillary biomarker panel. Exploratory analyses in the assay-complete mechanism-panel subset (*n* = 311). **(A)** Association between admission AT-III activity and thrombin–antithrombin complex concentration. **(B)** AT-III activity-to-antigen ratio across clinical severity strata. **(C)** Spearman correlation matrix of AT-III activity, AT-III antigen, activity-to-antigen ratio, thrombin–antithrombin complex, Protein C activity, free Protein S activity, soluble thrombomodulin, and ELANE mRNA fold change. Correlation *p* values were adjusted using the Benjamini–Hochberg false-discovery-rate procedure. AT-III activity was measured in fresh clinical samples, whereas antigen and ancillary analytes were measured in archived admission-day citrate plasma. Supporting data are reported in Supplementary Tables S18, S19.

In the 311-patient assay-complete mechanism-panel subset, AT-III antigen did not differ significantly across clinical severity strata (Kruskal–Wallis *p* = 0.102), with non-monotonic median values of 81.4, 83.3, 75.5, and 81.0% from infection/fever through septic shock. In contrast, AT-III activity declined from 82.0% in infection/fever to 58.0% in septic shock (*p* < 0.001). The derived activity-to-antigen ratio declined across severity strata from 0.99 to 0.94 to 0.86 to 0.70 (Kruskal–Wallis *p* < 0.001; Jonckheere–Terpstra *Z* = 12.0; Figure 8B; Supplementary Table S18).

The activity-to-antigen ratio correlated modestly with AT-III activity (Spearman *ρ* = 0.315) and inversely with AT-III antigen (*ρ* = −0.217), indicating that the ratio was not a simple rescaling of either component alone (Figure 8C; Supplementary Table S19). Storage duration differed across severity strata and remained associated with AT-III antigen after adjustment for severity (partial *ρ* = 0.257; *p* < 0.001), indicating that storage-related effects on antigen recovery cannot be excluded. Nevertheless, the severity–ratio association was materially unchanged after adjustment for storage duration (unadjusted Spearman *ρ* = −0.638; storage-adjusted partial *ρ* = −0.631; both *p* < 0.001). After adjustment for severity, storage duration was not associated with the ratio (partial *ρ* = 0.026; *p* = 0.65; Supplementary Figure S4; Supplementary Table S31).

Other measured markers also varied across severity strata. Protein C activity decreased from 95.6 to 36.9%, free Protein S activity decreased from 86.0 to 37.0%, and TAT increased from 5.00 to 39.11 ng/mL from infection/fever to septic shock (all *p* < 0.001; Figure 8; Supplementary Tables S18, S19). These findings are reported as exploratory biological context. Because AT-III activity was measured in fresh clinical samples whereas antigen and related analytes were measured retrospectively in archived admission-day plasma, the activity-to-antigen analysis does not establish a mechanism of AT-III dysfunction.

### 3.6 Selected MIMIC-IV direction-consistency analysis

In MIMIC-IV v3.1, 667 admissions had at least one AT-III activity measurement, including 51 coded septic-shock events defined by ICD-10 code R65.21. The minimal portable model including age, sex, and AT-III activity achieved an AUC of 0.864 (95% CI, 0.808–0.909; Supplementary Figure S5; Supplementary Table S21). The AT-III coefficient was negative (*β* = −1.35 per 1-SD increase), directionally compatible with the discovery-cohort association.

AT-III testing was selectively ordered in MIMIC-IV and occurred a median of 76 h after vasopressor initiation. Accordingly, this selected-cohort analysis provides cross-cohort direction consistency only and does not represent formal external validation of the discovery-cohort Firth models.

### 3.7 Supportive and exploratory analyses

Prespecified subgroup interaction analyses for the secondary severe-infection composite endpoint showed no statistically clear effect modification in six of the seven evaluated strata; a nominal interaction with the D-dimer stratum (interaction *p* = 0.018) was observed but was not treated as confirmatory, consistent with the prespecified descriptive, non-multiplicity-adjusted interpretation of subgroup analyses (Supplementary Figure S1; Supplementary Table S20). In 100 repeated 70/30 split-sample replications, the median held-out test-set AUC for Model 1b was 0.889 (IQR across replications, 0.870–0.910), with a mean of 18.3 septic-shock events in held-out test sets (Supplementary Table S15).

Hepatic-function sensitivity analyses, a clinically enriched infection-subgroup diagnostic sensitivity analysis, multiple imputation for the secondary composite endpoint, SIC-stratified partial-SOFA analyses, restricted cubic spline analyses, LASSO modelling, Bayesian estimation, and mechanism-panel two-marker modelling are reported in the Supplementary Tables S6, S8–S9, S11, S13, S15, S17, S24–S25; Supplementary Figures S1, S3, S4. Several of these analyses used the secondary severe-infection composite endpoint, partial-SOFA constructs, or sparse primary-event subsets; they were therefore interpreted as supportive or exploratory rather than confirmatory.

## 4 Discussion

In this retrospective infection-admission cohort, admission AT-III activity provided complementary risk-stratification information within the large subgroup below the ISTH 2001 overt-DIC threshold. Although 44 of 60 septic-shock events in the ISTH-complete cohort occurred in this subgroup, this predominance partly reflected the large size of the below-threshold stratum. Within that stratum, AT-III activity nevertheless further separated risk: 43 of 44 septic-shock cases had AT-III activity <80%, whereas the positive predictive value of this threshold was only 9.37%. Thus, the prespecified <80% threshold should be interpreted as a sensitivity-led enrichment signal rather than as a rule-in diagnostic test. Retrospective temporal adjudication further showed that AT-III report release preceded operational septic-shock onset in 62 incident cases by a median of 7.6 h. In the clinically indicated lactate-measured subset, AT-III also improved discrimination beyond age, sex, and lactate, including in the temporally ordered incident-shock analysis. Together, these findings support short-horizon admission-stage risk stratification, but they do not establish prospective prediction, workflow benefit, or stand-alone clinical decision-making.

The clinical contribution of the present study lies primarily in the setting examined rather than in a new analytical method. Previous studies have generally evaluated AT-III activity in patients already classified as having sepsis, sepsis-induced coagulopathy, or overt DIC, focusing on disease severity, mortality, or diagnostic classification within those established syndromes. Matsubara et al. related AT-III activity to 28-day mortality in the multicenter JAAM FORECAST cohort using spline modelling, without stratification by ISTH overt-DIC status or assessment of AT-III antigen (17). Wei et al. compared DIC scoring systems in patients with SIC-confirmed sepsis (18), Li et al. evaluated AT-III activity as a diagnostic marker for SIC in a prospective binary-classification setting (19), and Ren et al. reported prognostic associations of AT-III activity in MIMIC-III/IV cohorts (20). By contrast, the present analysis considered the admission-stage infection-to-shock spectrum and focused on risk enrichment among patients who did not meet the overt-DIC threshold. The comparison with the ISTH 2001 and 2025 SSC frameworks was contextual and limited to the septic-shock endpoint. Observed AUC differences should therefore not be interpreted as evidence that either ISTH framework performs poorly for its intended purpose of DIC assessment.

The exploratory activity-to-antigen analysis provides biological context but does not establish a mechanism of AT-III dysfunction. AT-III activity declined across clinical severity strata, whereas antigen values did not show a corresponding monotonic decline; consequently, the patient-level activity-to-antigen ratio decreased from 0.99 to 0.70. This pattern was not explained solely by absolute activity or antigen values and remained materially similar after accounting for storage duration. However, AT-III activity was measured in fresh clinical samples, whereas AT-III antigen, TAT, soluble thrombomodulin, and related analytes were measured retrospectively in archived admission-day citrate plasma that was not necessarily collected from the same blood draw. Incomplete recoverability of the prior freeze–thaw history further limits mechanistic interpretation. Experimental studies have suggested that neutrophil elastase-mediated cleavage may reduce AT-III activity under inflammatory conditions (13–15), but the present cross-sectional clinical observations cannot identify this or any other pathway as the dominant in-vivo explanation. Reduced hepatic synthesis, consumption, vascular leakage, oxidative modification, and residual pre-analytical variation remain plausible non-exclusive contributors. Similarly, parallel variation in Protein C, free Protein S, TAT, ELANE, and soluble thrombomodulin is compatible with coagulation activation, endothelial injury, and neutrophil activation in sepsis-associated coagulopathy, but does not isolate a single causal pathway.

The clinical interpretation of AT-III should remain deliberately conservative. AT-III activity <80% should not be regarded as a rule-in test, a treatment indication, or a standalone trigger for escalation of care. In the ISTH below-threshold stratum, 416 of 459 admissions with AT-III activity <80% did not have septic shock, underscoring the limited specificity of this threshold. Where AT-III is already included in the local admission coagulation workflow, it may be available without additional patient sampling and could be evaluated prospectively as one candidate anticoagulant-pathway signal within a structured reassessment strategy. Such a strategy would retain clinical assessment, standard sepsis evaluation, and ISTH-based DIC assessment rather than replacing them. The present findings concern diagnostic enrichment only and should not be interpreted as support for therapeutic AT-III supplementation, for which the broader clinical literature reports mixed or uncertain findings (34–38).

Several design features strengthen the interpretation of the discriminative findings. First, the septic-shock reference endpoint did not directly include AT-III activity or the raw ISTH score components, reducing direct component overlap between the index marker, comparator framework, and outcome. This does not eliminate confounding by overall illness severity, but it avoids the direct circularity that can arise when AT-III is evaluated against SIC-defined endpoints incorporating overlapping hemostatic variables. Second, the study explicitly distinguished incident shock after AT-III report release from prevalent or concurrent shock, rather than treating all septic-shock cases as temporally equivalent. Third, the association of lower AT-III activity with septic shock remained evident in Firth-penalized modelling after adjustment for age, sex, and raw ISTH components; bootstrap correction and calibration assessment were also performed. Finally, selected lactate analyses suggested that AT-III contributed discrimination beyond age, sex, and lactate in patients with clinically measured lactate values, although this comparison should not be interpreted as comprehensive adjustment for global severity.

Several limitations are important. The primary below-threshold analysis included only 44 septic-shock events, and the unified Firth model included 58 events; penalized estimation, bootstrap correction, and calibration analyses can reduce but cannot remove sparse-event uncertainty. Full SOFA, APACHE II, and uniformly measured lactate values were unavailable. Accordingly, the conditional association observed beyond raw ISTH components, and the selected-subset association observed beyond age, sex, and lactate, should not be interpreted as independence from global illness severity. Structured clinical gestalt and the individual qSOFA components (respiratory rate, altered mentation, and systolic blood pressure) could not be reliably or completely reconstructed from the retrospective record; selected clinically indicated lactate was therefore the only routinely documented bedside marker against which AT-III could be benchmarked in this cohort. A head-to-head comparison of AT-III with qSOFA, full SOFA, and clinician gestalt will require prospective, time-stamped data collection. Lactate testing was clinically indicated rather than uniformly performed, and lactate contributes to the operational septic-shock definition; its analysis therefore provides a pragmatic comparison with a commonly used bedside marker rather than a severity-adjusted causal estimate. AT-III availability also reflected routine-care testing, with valid admission-window results available in 1,187 of 1,286 eligible admissions; the comparison with AT-III-unmeasured admissions reduces concern about major measured differences but cannot exclude selection associated with testing practices. Likewise, non-uniform availability of the four ISTH components may have affected the composition of the ISTH-complete and model-specific complete-case subsets; component-specific missingness by clinical-severity stratum is reported in Supplementary Table S26, but residual selection associated with laboratory availability cannot be excluded. In an exploratory subgroup analysis, the inverse association between AT-III activity and the composite endpoint appeared stronger among admissions with elevated D-dimer (interaction *p* = 0.018, uncorrected); this hypothesis-generating signal warrants prospective confirmation.

The temporal analysis also has intrinsic limitations. It used AT-III report-release time rather than exact sample-collection-to-onset lead time and operationalized shock onset as the earliest time at which both vasopressor treatment and lactate >2 mmol/L were documented. This retrospective reconstruction supports temporal ordering in the incident cases but does not establish prospective workflow performance, the true biological lead time of AT-III decline, or improved clinical outcomes after acting on the result. The mechanism panel was *post hoc* and based on fresh activity measurements paired at the patient level with retrospectively tested archived admission-day plasma, leaving residual pre-analytical uncertainty despite storage-duration sensitivity analyses. The MIMIC-IV analysis was restricted to a selectively tested AT-III cohort, used coded septic shock rather than full Sepsis-3 ascertainment, and measured AT-III a median of 76 h after vasopressor initiation; it therefore provides direction consistency only, not formal external validation. Finally, this was a single-center Chinese tertiary-care cohort restricted to adults aged 18–70 years according to the source-cohort eligibility framework. This restriction is a material limitation for the proposed admission-screening use case, because patients older than 70 years carry the highest sepsis and septic-shock burden yet also more frequently have non-coagulopathic causes of reduced AT-III activity—including impaired hepatic synthesis, malnutrition, and age-related differences in coagulation physiology—that could attenuate the specificity of a low-AT-III signal. The present discrimination estimates should therefore not be assumed to transfer to geriatric or general ICU populations, and age-stratified prospective evaluation across the full adult age range will be required before any screening application.

Future work should prospectively evaluate whether AT-III can contribute to a clinically useful reassessment strategy in broader infection cohorts. A multicenter study should collect time-stamped blood-draw and report-release times, serial lactate measurements, full SOFA or APACHE II data, vasopressor initiation, ICU transfer, and patient-important outcomes, including time to septic-shock recognition and short-term mortality. Mechanistic work should use prospectively matched fresh specimens from the same blood draw to measure AT-III activity, antigen, TAT, endothelial markers, and candidate protease activity, ideally complemented by proteomic identification of AT-III cleavage products. External confirmation in cohorts with contemporaneous coagulation and inflammatory measurements will be necessary before any mechanism-informed extended model or AT-III-guided clinical workflow can be considered for deployment.

## 5 Conclusion

Admission AT-III activity was associated with septic shock within the large ISTH 2001 below-threshold infection subgroup and provided high-sensitivity but low-positive-predictive-value enrichment information. These findings are limited to adults aged 18–70 years in a single-center cohort. In incident cases, AT-III report release preceded operational shock onset, and selected-lactate analyses suggested incremental discrimination beyond age, sex, and lactate. These findings support AT-III as a complementary anticoagulant-pathway marker for retrospective diagnostic enrichment rather than as a rule-in diagnostic test, treatment trigger, standalone prediction tool, or replacement for ISTH-based DIC assessment. Prospective, time-stamped multicenter studies with comprehensive severity assessment and patient-important outcomes are required before clinical deployment.

## Data Availability

The original contributions presented in the study are included in the article/Supplementary material, further inquiries can be directed to the corresponding author/s.
