# Supplementary material for: Admission antithrombin III activity as a risk-enrichment marker for septic shock below the ISTH overt-DIC threshold
Source: Front Med (Lausanne). 2026 Jul 31;13:1899792. doi: 10.3389/fmed.2026.1899792 (PMC13473283; doi:10.3389/fmed.2026.1899792)
Supplement: Supplementary file 1 [file Table_1.DOCX]

**Supplementary Material**

**Contents**

Part 1. Supplementary Methods

§S2.5. ISTH 2001 overt-DIC and 2025 SSC overt-DIC scoring rules

§S2.7. Mechanism panel: multi-platform assay details and quality control

§S2.9. Statistical analysis — exploratory analyses

§S2.9.2 RCS dose-response; §S2.9.3 LASSO; §S2.9.4 Bayesian posterior; §S2.9.5 NRI/IDI and decision-curve analysis

§S2.9.6. SIC-stratified partial-SOFA secondary analysis

Part 2. Supplementary Figure Legends (sFig1–sFig5)

Part 3. Supplementary Table Index (sTableS1–sTableS35, distributed across Workbooks A–D)

**Part 1. Supplementary Methods**

**§S2.5 ISTH 2001 overt-DIC and 2025 SSC overt-DIC scoring rules**

The ISTH 2001 overt-DIC score is the sum of points across four components: platelet count (>100 = 0; 50–100 = 1; <50 = 2), prolongation of prothrombin time (<3 s = 0; 3–6 s = 1; >6 s = 2; we used INR with the conventional substitution <1.3 = 0, 1.3–1.7 = 1, >1.7 = 2 owing to inconsistent direct PT prolongation reporting in the source records), fibrinogen (>1 g/L = 0; ≤1 g/L = 1), and a fibrin-related marker (D-dimer: no increase = 0, moderate = 2, strong = 3). Overt DIC was defined as a total score of 5 or greater.

The ISTH 2025 SSC overt-DIC score is the sum of points across four components: platelet count (≥100 × 10⁹/L = 0; 50–<100 × 10⁹/L = 1; <50 × 10⁹/L = 2), D-dimer expressed relative to the assay-specific upper limit of normal (≤3 × ULN = 0; >3–≤7 × ULN = 2; >7 × ULN = 3), prothrombin-time prolongation (<3 s = 0; ≥3–<6 s = 1; ≥6 s = 2), and fibrinogen (≥1.0 g/L = 0; <1.0 g/L [<100 mg/dL] = 1). Overt DIC was defined as a total score of 5 or greater, per Iba et al. 2025 (8). D-dimer was reported in ng/mL fibrinogen-equivalent units on a single platform throughout the study period, with an upper limit of normal of 500 ng/mL; the 3× and 7× thresholds therefore corresponded to 1,500 and 3,500 ng/mL. Because the source records did not include a separately reported prothrombin-time control value, prothrombin-time prolongation in seconds was recovered for each admission from the measured prothrombin time and international normalized ratio as PT − PT/INR (equivalent to assuming an International Sensitivity Index of approximately 1), yielding a stable reference prothrombin time of approximately 11.1 s. All four components were required, so the 2025 SSC overt-DIC score was computable in the same score-complete subset as the ISTH 2001 score (n = 1,027).

Inter-framework agreement on overt-DIC classification was quantified using Cohen's κ on the shared subset.

**§S2.7 Mechanism panel: multi-platform assay details and quality control**

The post-hoc mechanism panel combined analytes from three sources. AT-III antigen, thrombin–antithrombin (TAT) complex, and soluble thrombomodulin (sTM) were measured retrospectively by sandwich ELISA on archived citrate plasma collected at admission and stored at −80 °C until a single batch analysis on 30 June 2025. Protein C activity and free Protein S activity were routine clinical coagulation-platform measurements obtained at admission. ELANE (neutrophil elastase) mRNA was quantified by qRT-PCR. Analytical quality control was therefore platform-specific.

**Sandwich ELISA (AT-III antigen, TAT, sTM): assays used four-parameter-logistic calibration. Final analytical runs were assessed by spike recovery (acceptance 80–120%) and duplicate relative difference (acceptance <15%); observed recovery and duplicate-difference ranges by assay are summarised in Supplementary Table S32, with run-level records in Supplementary Table S34. The AT-III antigen immunoassay recognises an epitope outside the reactive-site loop and detects both intact and proteolytically cleaved AT-III.**

**Coagulation platform (Protein C, Protein S): Protein C activity (chromogenic assay using a snake-venom activator and a synthetic p-nitroaniline substrate read at 405 nm) and free Protein S activity (clotting-based assay using activated Protein C as cofactor in a modified APTT reaction) were obtained as routine clinical coagulation-laboratory tests on the same analyser used for admission AT-III activity (Sysmex CS-series), under the laboratory’s routine internal quality control. These two analytes were not part of the post-hoc ELISA batch and are not included in the ELISA-specific QC summary (Supplementary Table S32).**

**Quantitative real-time RT-PCR (ELANE): ELANE mRNA fold-change was quantified by 2-step qRT-PCR with ACTB as the reference gene. RNA was extracted from whole-blood specimens using a silica-based spin-column protocol with on-column DNase digestion (260/280 ratios 1.9–2.1); reverse transcription used random hexamer priming; amplification used SYBR Green chemistry in technical duplicate. Run-level quality control comprised primer-efficiency estimation, melt-curve single-peak review, and review of no-template and no-reverse-transcriptase controls; primer-efficiency and melt-temperature summaries are provided in Supplementary Table S33, with run records in Supplementary Table S34.**

The 311-patient mechanism subset comprises admissions enrolled before the June 2025 batch-processing date with quality-control-qualified analytical results. The panel is reported as exploratory biological plausibility rather than as in-vivo mechanism evidence. Complete pre-analytical freeze–thaw history before batch testing was not uniformly recoverable from source records; storage-duration sensitivity analyses addressing this residual uncertainty are reported in Supplementary Table S31.

**§S2.9 Statistical analysis — exploratory analyses**

**§S2.9.2 Restricted cubic spline (RCS) dose–response**

AT-III activity was modelled with a 4-knot restricted cubic spline at the 5th, 35th, 65th, and 95th percentiles per Harrell (33). Overall and non-linearity Wald tests were reported separately. Both the primary septic-shock endpoint and the secondary severe-infection composite endpoint were modelled; RCS statistics are provided in sTableS17 (the exploratory dose-response figure was not retained).

**§S2.9.3 LASSO variable selection**

As an exploratory penalised-selection sensitivity analysis, L1-penalised (LASSO) logistic regression was fit across 59 candidate clinical, demographic, laboratory and severity variables. The penalty was selected by 10-fold cross-validation, and the variables retained at the cross-validation-optimal penalty are reported in Supplementary Table S8. This analysis was exploratory and did not define the primary model; the LASSO-selected variable set achieved cross-validated discrimination comparable to a manually specified clinical model.

**§S2.9.4 Bayesian posterior estimation**

Bayesian posterior estimation was applied to the exploratory two-marker mechanism-panel model (AT-III activity + TAT) using PyMC 5.15 with weakly-informative N(0, 2.5) priors on both the standardised coefficients and the intercept. Four NUTS chains of 1,000 warm-up + 2,000 sampling iterations were run; convergence was confirmed by R-hat < 1.01 and effective sample size > 1,500 for all parameters. Posterior median AUC and 95% highest-density interval (HDI) are reported in sTableS13 section C.

**§S2.9.5 NRI, IDI, and decision-curve analysis (DCA)**

Net Reclassification Improvement (NRI) was computed using the category-free (continuous) variant per Pencina et al. (30), with Kerr et al.'s (31) interpretive guidance on event/non-event decomposition. Integrated Discrimination Improvement (IDI) was computed per Pencina et al. (30). For NRI and IDI, the base model comprised the four raw ISTH components—platelet count, INR, fibrinogen, and D-dimer—and the augmented model additionally included AT-III activity. Demographic covariates were not included in these reclassification analyses.

For decision-curve analysis, the base model comprised the raw ISTH components only—platelet count, INR, fibrinogen, and D-dimer—without demographic covariates; the augmented model additionally included AT-III activity. Decision curves were displayed across threshold probabilities of 0–50%, with interpretation focused on clinically plausible thresholds of 5–30%. Both primary septic-shock and secondary severe-infection composite reclassification analyses are reported in Supplementary Table S12. Primary septic-shock NRI/IDI estimates are exploratory because of event sparsity and the recognised interpretive limitations of reclassification metrics; secondary severe-infection composite estimates are supportive. Decision-curve analysis for the primary septic-shock endpoint within the ISTH below-threshold subgroup is shown in Figure 8B, whereas the secondary severe-infection composite analysis is shown in Supplementary Figure S2. These analyses address different endpoints and clinical questions.

**§S2.9.6 SIC-stratified partial-SOFA secondary analysis**

The ISTH 2017 sepsis-induced coagulopathy (SIC) framework combines three items: platelet count item (>150 × 10⁹/L = 0, 100–150 = 1, <100 = 2), INR item (≤1.2 = 0, 1.2–1.4 = 1, >1.4 = 2), and total SOFA score item (0 = 0, 1 = 1, ≥2 = 2). SIC positivity is defined as total SIC score of 4 or greater AND coagulation subtotal (PLT item + INR item) of 2 or greater.

Because the source records did not uniformly capture all six SOFA components, partial SOFA was computed from three components routinely available at admission: hepatic SOFA (from serum total bilirubin in µmol/L, with conventional cutpoints), renal SOFA (from serum creatinine in µmol/L), and a cardiovascular surrogate (assigned 3 in patients with septic shock (Sepsis-3 criteria), 0 otherwise). Respiratory, hematology, and CNS SOFA components were not available and were imputed at 0, which conservatively biases partial-SOFA scores downward and SIC classification toward SIC-negative.

The SIC-computable subset is defined as patients with PLT + INR + bilirubin + creatinine + AT-III activity all available (n = 835). Within this subset, SIC × AT-III (< 80 vs ≥ 80%) stratification of the secondary severe-infection composite endpoint is reported in sTableS24 with risk ratios and Wilson 95% CIs; sensitivity to partial-SOFA component choice and SIC threshold is reported in sTableS25; and the 4-bar visualisation is provided as Supplementary Figure S3. Because SIC contains organ-dysfunction components shared with the Sepsis-3 sepsis endpoint, this analysis is reported as a supportive framework analysis rather than an independent sepsis-diagnosis evaluation.

**Part 2. Supplementary Figure Legends**

**Supplementary Figure 1. ISTH strata and subgroup interaction analyses.**

Panel A: AT-III activity across ISTH 2001 DIC-score strata. The upper panel shows median AT-III activity with interquartile ranges, and the lower panel shows the prevalence of AT-III activity <80%, across ISTH scores 0–1, 2–3, 4, and ≥5. Panel B: forest plot of subgroup × AT-III interaction tests across seven strata (ISTH overt-DIC status, platelet count, NLR, D-dimer, fibrinogen, age, sex), with stratum-specific odds ratios (95% CI) and likelihood-ratio P values for the interaction term. All interactions returned P > 0.15.These subgroup-interaction analyses used the secondary severe-infection composite endpoint and are descriptive and exploratory; no multiplicity-adjusted confirmatory inference was performed.

**Supplementary Figure 2. Exploratory decision-curve analyses for the secondary severe-infection composite endpoint.**

The decision-curve analysis compares a four-component ISTH base model comprising platelet count, INR, fibrinogen, and D-dimer with the corresponding model additionally including AT-III activity, without demographic covariates. The y-axis shows net benefit, and the x-axis displays threshold probabilities from 0% to 50%; interpretation focused on clinically plausible thresholds of 5–30%. Treat-all and treat-none reference curves are overlaid. This secondary severe-infection composite decision-curve analysis is exploratory. A separate primary septic-shock decision-curve analysis within the ISTH below-threshold subgroup is shown in Figure 8B.

**Supplementary Figure 3. SIC-stratified partial-SOFA secondary analysis.**

Sepsis-level illness rate within the four cells of the SIC (positive vs negative) × AT-III (< 80% vs ≥ 80%) stratification, in the SIC-computable subset (n = 835). Bars show event rates with 95% Wilson confidence intervals; numerical labels show events/n. Within the SIC-negative stratum (n = 770), AT-III < 80% raised event rate from 6.3% (26/414) to 18.0% (64/356), a risk ratio of 2.86. Partial-SOFA was computed from three of six SOFA components routinely available at admission (hepatic, renal, cardiovascular surrogated by septic-shock status); respiratory, hematology, and CNS components were imputed at 0. The dotted reference line marks the overall sepsis-level illness rate (15.9%) in the SIC-computable subset. This analysis is supportive rather than an independent sepsis-diagnosis evaluation, because SIC components overlap with the Sepsis-3 endpoint construct.

**Supplementary Figure 4. Storage-duration sensitivity of the exploratory AT-III activity-to-antigen ratio.**

Sensitivity of the severity-associated decline in the AT-III activity-to-antigen ratio to biobank storage duration, in the 311-patient mechanism panel (single ELISA assay date 30 June 2025). (A) Storage duration (days) across the four infection-severity strata; (B) activity-to-antigen ratio across the same strata; (C) correlation-coefficient forest plot (one unadjusted Spearman correlation and three partial correlations): the severity–ratio association is essentially unchanged after adjustment for storage duration (unadjusted r = −0.638 versus storage-adjusted r = −0.631), whereas storage duration is only weakly related to the ratio once severity is accounted for (r = 0.026, P = 0.65). Storage duration also remained associated with AT-III antigen after adjustment for severity (r=0.257, P<0.001), indicating that storage-related effects on antigen recovery cannot be excluded. However, the severity–ratio association changed minimally after adjustment for storage duration, and storage duration was not associated with the ratio after adjustment for severity. These analyses reduce, but do not eliminate, residual pre-analytical uncertainty.

**Supplementary Figure 5. MIMIC-IV direction-consistency analysis.**

Cross-cohort direction-consistency check in MIMIC-IV v3.1: 667 admissions with at least one antithrombin activity measurement (itemid 51140) over 2008–2022, including 51 ICD-10 R65.21 septic-shock events (616 non-shock admissions). (A) Receiver-operating-characteristic curve for a minimal portable model (age, sex, AT-III activity), plotted from the actual model-predicted probabilities (AUC 0.864, 95% CI 0.808–0.909; AT-III coefficient β = −1.35 per 1-SD activity increase). (B) AT-III activity distribution in shock (n = 51; median 45%) versus non-shock (n = 616; median 88%) admissions (Mann–Whitney P < 0.001). Because AT-III testing in MIMIC-IV is ordered selectively (a median of 76 h after vasopressor initiation), this analysis is a direction-consistency check rather than formal external validation (Supplementary Table S21).

**Part 3. Supplementary Table Index**

Thirty-five supplementary tables are organised into four thematic Excel workbooks so that each table retains its sTable number as a sheet name and can be cited directly from main-text "Supplementary Table SX" references. Each workbook contains a Contents sheet that lists its sheets. Workbook assignment is summarised below.

**Workbook A — Cohort and threshold analyses**

**sTableS1.** STROBE flow diagram with per-stage exclusions and retained N (Workbook A)

**sTableS2.** Marker cutoff sensitivity (D-dimer and AT-III cutpoints by endpoint) (Workbook A)

**sTableS3.** Subset-vs-full-cohort representativeness for the AT-III–available and ISTH-complete subsets (Workbook A)

**sTableS4. Comparison of the ISTH 2001 and 2025 SSC overt-DIC scores for the septic-shock endpoint (Workbook A)**

**sTableS5. Robustness of AT-III discrimination across ISTH overt-DIC thresholds (Workbook A)**

**sTableS6.** Clinically enriched bacteremia/sepsis/shock subgroup — diagnostic performance (Workbook A)

**sTableS26. Missingness of ISTH component variables by infection-severity stratum (Workbook A)**

**sTableS27. Reconciliation of analytical denominators and septic-shock event counts (Workbook A)**

**sTableS29. De-identified temporal and lactate analysis dataset (n = 1,296) (Workbook A)**

**sTableS30. AT-III activity versus serum lactate: head-to-head discrimination and incremental value for septic shock, within the lactate-measured subset (Workbook A)**

**Workbook B — Models and validation**

**sTableS7.** Calibration and goodness-of-fit (Hosmer–Lemeshow, calibration slope, Brier) (Workbook B)

**sTableS8. LASSO-penalised variable selection (exploratory sensitivity analysis; Workbook B)**

**sTableS9.** MICE multiple-imputation pooled summary (secondary composite endpoint) (Workbook B)

**sTableS10.** Bootstrap optimism correction at the primary Youden cutpoint (69.0%) (Workbook B)

**sTableS11.** Model 2 coefficients with hepatic-sensitivity variant (Model 2b adding albumin) (Workbook B)

**sTableS12. NRI / IDI / ΔAUC / ΔAIC reclassification metrics for the primary septic-shock and secondary composite endpoints (Workbook B)**

**sTableS13. Exploratory clinical and mechanism-panel model diagnostics (secondary composite + two-marker AT-III + TAT) (Workbook B)**

**sTableS14. Firth-penalised septic-shock model performance spine (Models 1a, 1b, 2; unified complete-case subset) (Workbook B)**

**sTableS15.** Split-sample 70/30 internal validation — median test-set AUC over 100 replicates (Workbook B)

**sTableS16. Primary Model 1b coefficients with centring means and SDs (Workbook B)**

**Workbook C — Mechanism and dose-response**

**sTableS17.** Dose-response statistics: RCS + quartile trend tests for AT-III activity and activity/antigen ratio (Workbook C)

**sTableS18.** Mechanism markers by infection severity group (n = 311; medians, IQR, Kruskal–Wallis P) (Workbook C)

**sTableS19.** Spearman correlation matrix among the eight mechanism panel variables (n = 311) (Workbook C)

**sTableS31. Storage-duration sensitivity of the exploratory AT-III activity-to-antigen ratio (Workbook C)**

**sTableS32. Analytical quality-control summary for post-hoc ELISA assays (recovery, duplicate) (Workbook C)**

**sTableS33. Run-level quality-control summary for ELANE qRT-PCR (primer efficiency, melt temperature) (Workbook C)**

**sTableS34. Run-level quality-control records for post-hoc ELISA and ELANE qRT-PCR (Workbook C)**

**sTableS35. De-identified mechanism-panel dataset (n = 311) (Workbook C)**

**Workbook D — External, subgroup, and secondary analyses**

**sTableS20.** Subgroup × AT-III interaction analyses on the secondary severe-infection composite endpoint (Workbook D)

**sTableS21.** MIMIC-IV pilot — minimal portable model directional consistency (n = 667 admissions, 51 events) (Workbook D)

**sTableS22.** Single-marker discriminative AUCs by endpoint, AT-III–available subset (replaces former Table 3b) (Workbook D)

**sTableS23.** DeLong paired AUC comparisons against AT-III activity by endpoint (replaces former Table 3c) (Workbook D)

**sTableS24.** SIC × AT-III stratification of sepsis-level illness risk, SIC-computable subset (n = 835) (Workbook D)

**sTableS25.** SOFA-component sensitivity analysis — enrichment robustness across partial-SOFA definitions and SIC thresholds (Workbook D)

**sTableS28. Temporal adjudication of septic-shock relative to AT-III result availability (Workbook D)**

*Cross-references in the main manuscript point directly to these sheet names; the Contents sheet in each workbook duplicates this index.*
